# ADAR1-mediated RNA editing links ganglioside catabolism to glioblastoma stem cell maintenance

**DOI:** 10.1172/JCI143397

**Published:** 2022-03-15

**Authors:** Li Jiang, Yajing Hao, Changwei Shao, Qiulian Wu, Briana C. Prager, Ryan C. Gimple, Gabriele Sulli, Leo J.Y. Kim, Guoxin Zhang, Zhixin Qiu, Zhe Zhu, Xiang-Dong Fu, Jeremy N. Rich

**Affiliations:** 1 Division of Regenerative Medicine, Department of Medicine, UCSD, La Jolla, California, USA.; 2 Sanford Consortium for Regenerative Medicine, La Jolla, California, USA.; 3 Department of Cellular and Molecular Medicine, UCSD, La Jolla, California, USA.; 4 UPMC Hillman Cancer Center, Pittsburgh, Pennsylvania, USA.; 5 Medical Scientist Training Program,; 6 Cleveland Clinic Lerner College of Medicine, and; 7 Department of Pathology, Case Western Reserve University, Cleveland, Ohio, USA.; 8 Department of Neurology, University of Pittsburgh, Pittsburgh, Pennsylvania, USA.

**Keywords:** Oncology, Stem cells, Brain cancer, Human stem cells

## Abstract

Glioblastoma (GBM) is the most common and lethal primary malignant brain tumor, containing GBM stem cells (GSCs) that contribute to therapeutic resistance and relapse. Exposing potential GSC vulnerabilities may provide therapeutic strategies against GBM. Here, we interrogated the role of adenosine-to-inosine (A-to-I) RNA editing mediated by adenosine deaminase acting on RNA 1 (ADAR1) in GSCs and found that both ADAR1 and global RNA editomes were elevated in GSCs compared with normal neural stem cells. ADAR1 inactivation or blocking of the upstream JAK/STAT pathway through TYK2 inhibition impaired GSC self-renewal and stemness. Downstream of ADAR1, RNA editing of the 3′-UTR of GM2A, a key ganglioside catabolism activator, proved to be critical, as interference with ganglioside catabolism and disruption of ADAR1 showed a similar functional impact on GSCs. These findings reveal that RNA editing links ganglioside catabolism to GSC self-renewal and stemness, exposing a potential vulnerability of GBM for therapeutic intervention.

## Graphical abstract

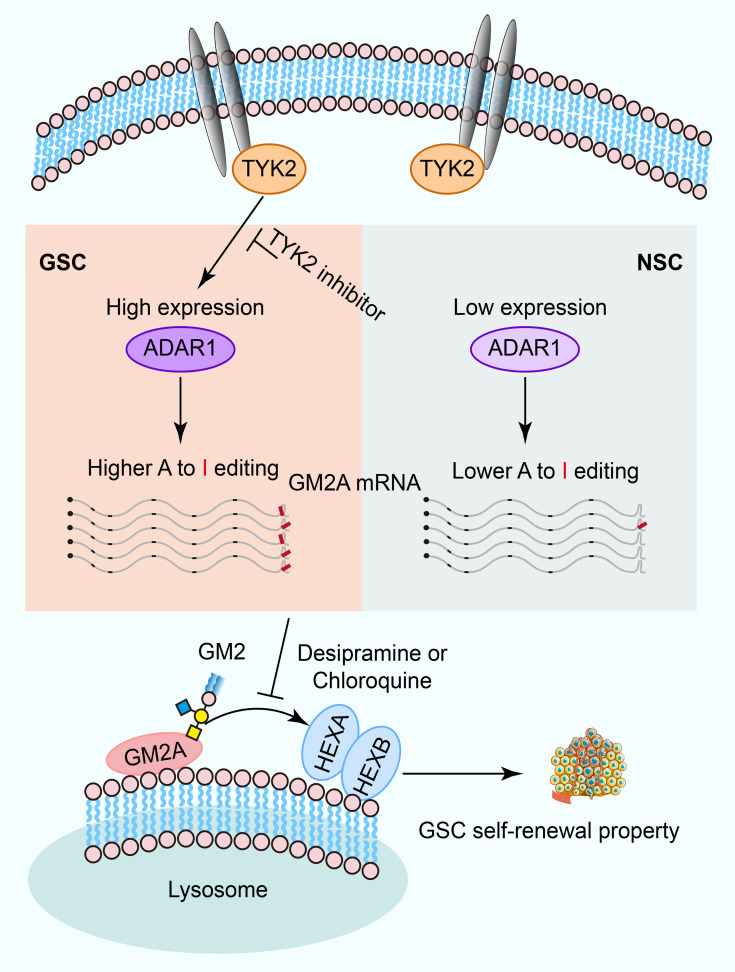

## Introduction

Glioblastomas (GBMs; World Health Organization grade IV gliomas) are the most prevalent and aggressive primary malignant intrinsic brain tumors in adults (1). GBMs remain universally fatal despite maximal surgical resection followed by chemoradiotherapy and adjuvant chemotherapy (2–4). GBMs display remarkable cellular heterogeneity, containing stem-like GBM stem cells (GSCs; also known as brain tumor–initiating cells) that contribute to therapeutic resistance and rapid recurrence (5–8). In contrast to non-stem or differentiated GBM cells (DGCs), GSCs express stem cell markers, generate spheres in serum-free conditions, and rapidly form tumors in vivo (9, 10). Somatic mutations contribute to initiation and progression of GBM, but precision medicine has so far met with limited success in its treatment (11, 12). Epigenetic alterations may also promote gliomagenesis, thus offering therapeutic targets (13–15). One recent advance in tumor biology is the attribution of altered A-to-I RNA editing to diverse tumorigenic pathways (16, 17).

In mammals, RNA editing alters transcript sequences of expressed RNA without affecting DNA sequences (18–20). A-to-I RNA editing, catalyzed by ADARs (adenosine deaminases acting on RNA), is the most common RNA editing event in mammals, with more than 85% of RNAs likely to be edited in coding and/or noncoding regions (19, 21). Three enzymes perform A-to-I RNA editing: ADAR1, ADARB1 (ADAR2), and ADARB2 (ADAR3) (22, 23). ADAR1 and ADARB1 have editing enzyme activities, whereas ADARB2 serves a regulatory role. ADARB1 acts on coding regions, while ADAR1 editing occurs mostly within double-stranded RNA (dsRNA) loops formed by inverted Alu repetitive elements (23, 24). Inosine is recognized as guanine, thereby affecting codon usage (25), alternative splicing (26), RNA stability, and function of various regulatory RNAs, including microRNAs (27–29). Thus, RNA editing enriches transcriptomic and phenotypic diversity (21, 30).

Dysregulation of RNA editing enzymes and editing frequency is commonly observed in cancers. In different tumor contexts, such dysregulation promotes tumor development, as in esophageal cancer (31) and gastric cancer (32), or causes tumor suppression, as in metastatic melanomas and astrocytoma (33, 34). One of the challenges in understanding oncogenic or tumor-suppressive functions of dysregulated RNA editing is to identify a specific editing event(s) and its connection to a critical biological pathway(s). In prostate cancer, A-to-I RNA editing alters the interaction of androgen receptor with androgens or anti-androgenic ligands (35). In liver cancer, editing of the antizyme inhibitor AZIN1 induces its cytoplasmic-to-nuclear translocation to increase tumor aggressiveness (36). In colorectal cancer, A-to-I RNA editing impacts Ras homolog family member Q (RHOQ) to promote invasion (37).

In GBM, ADARB1-editing activity has been suggested to have a role in tumor suppression (38), but dysregulation of different ADARs in GSCs remains largely unknown, and the effects of specific altered editing event(s) that may functionally contribute to brain tumor development have not been identified. We herein report that ADAR1 is the major RNA editing enzyme dysregulated in GSCs. ADAR1 upregulation confers GSC self-renewal and stemness, and one of its key substrates is GM2A, a critical activator involved in activating ganglioside catabolism. We further exploit this vulnerability in brain cancer and show that specific small-molecule inhibitors against key components in this pathway efficiently block GSC self-renewal and stemness, thus suggesting a potential therapeutic strategy against GBM.

## Results

### Global landscapes of A-to-I RNA editing in GSCs.

**Figure 1:**
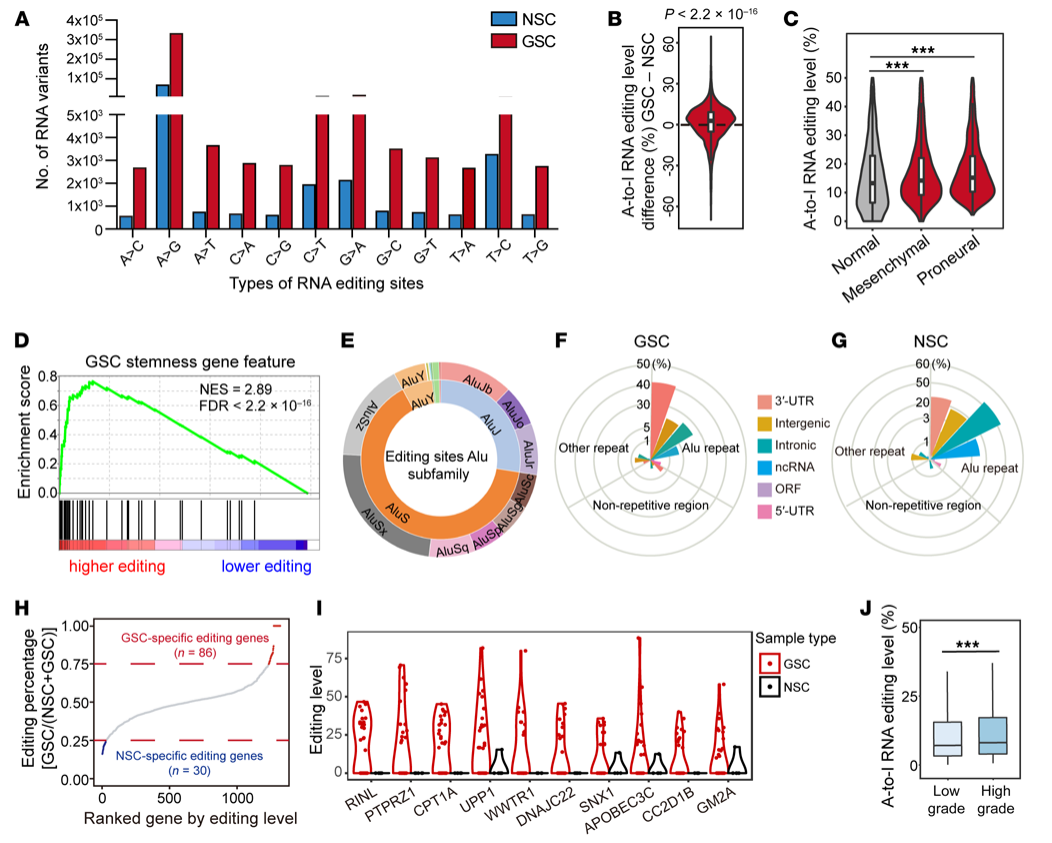
Global landscapes of A-to-I RNA editing in GSCs. See also Supplemental Figure 1. (**A**) Distribution of 12 types of RNA editing events in GBM stem cells (GSCs) and neural stem cells (NSCs). Data represent the mean number of inferred RNA editing events detected in GSCs and NSCs. (**B**) The comparative distribution of A-to-I RNA editing levels in GSCs relative to NSCs. *P* value was determined by Wilcoxon’s signed-rank test. (**C**) The distribution of A-to-I RNA editing levels in normal NSCs and different subtypes of GSCs. Statistical significance was determined by Wilcoxon’s signed-rank test and adjusted using the Bonferroni method. ***P < 0.001. (**D**) Gene set enrichment analysis of GSC stemness gene feature with different A-to-I RNA editing patterns. (**E**) The distribution of A-to-I RNA editing events in different types of Alu subfamilies. (**F**) The distribution and percentage of A-to-I RNA editing events in different regions of RNA transcripts in GSCs. (**G**) The distribution and percentage of A-to-I RNA editing events in different regions on RNA transcripts in NSCs. (**H**) The distribution of editing percentage of genes ranked by editing level. Eighty-six genes were defined as having GSC-specific editing, and 30 genes were defined as having NSC-specific editing. (**I**) The 10 genes, among those defined in **H** as having GSC-specific editing, with the highest levels of editing and dysregulation in cancer biology are depicted. (**J**) Relative frequency of A-to-I RNA editing events in low-grade and high-grade gliomas in TCGA (17). Statistical significance was determined by Wilcoxon’s signed-rank test. ***P < 0.001.

To elucidate A-to-I RNA editing in GSCs, we interrogated the RNA editomes of 31 patient-derived GSCs and 5 neural stem cells (NSCs) by RNA-Seq (39), using a previously described computational pipeline (refs. 17, 40, 41; and Supplemental Figure 1A; supplemental material available online with this article; https://doi.org/10.1172/JCI143397DS1). To eliminate false positives resulting from potential DNA contamination, we matched the existing whole exome sequencing data from this cohort of GSCs. We examined the distribution of 12 well-known types of RNA variants, revealing that A-to-G variants were 30–70 times higher than other variants in both GSCs and NSCs (Figure 1A), which is consistent with previous reports showing that A-to-I RNA editing is the most prevalent type of RNA editing events in human cells (42–44).

Next, we detected and prioritized candidate editing sites based on prevalence in at least 10 samples that showed high sequencing coverage for subsequent analyses. In total, we detected 6514 high-confidence editing sites. Further analysis of the distribution of RNA editing events across our samples revealed higher editing levels in GSCs compared with NSCs (Figure 1B), especially in the proneural transcriptional subtype (Figure 1C), suggesting a potential oncogenic role of elevated RNA editing in GBM. Gene ontology of genes with higher editing levels in GSCs compared with NSCs included regulation of stem cell differentiation, DNA replication, and RNA metabolism (Supplemental Figure 1, B and C). Gene set enrichment analysis showed that genes with highly enriched A-to-I RNA editing had a GSC signature, suggesting a role in stemness of the high editing in GSCs (Figure 1D). As expected from prior localization of A-to-I landscapes in human cells (45, 46), A-to-I–edited sites were preferentially localized to Alu repetitive elements, especially in the AluS subfamily (Figure 1E). GSC-enriched editing events were twice as likely to be enriched in 3′-UTR regions compared with intronic or intergenic regions (Figure 1F), whereas the converse trend was apparent with NSC-specific edits, suggesting that A-to-I RNA editing may be more important in the fate of these specific genes in GSCs relative to NSCs (Figure 1G), including the regulation of gene splicing and expression.

The majority of GSC-enriched editing events displayed highly conserved genomic localization in primates (Supplemental Figure 1D); however, comparison of these sites with those in other cancer types revealed less than 1% overlap (Supplemental Figure 1E), suggesting a potential unique contribution of GSC-enriched A-to-I editing events to GBM etiology and/or progression. Eighty-six genes were specifically edited in GSCs (Figure 1H), a fraction of which showed dominant editing events in comparison with NSCs. The genes with the highest fraction of sites edited and dysregulated in cancer biology are depicted in Figure 1I, including *PTPRZ1*, which is a marker of radial glia and GSCs (47). Analysis of The Cancer Genome Atlas (TCGA; ref. 17) revealed that editing levels positively correlated with GBM grade (Figure 1J) and negatively correlated with patient survival in selected genes (Supplemental Figure 1, F–J). Selected editing sites that we identified were distinct from previous reports (17), suggesting that differences may be derived from distinct sequencing depth, intertumoral heterogeneity, or other factors, but also reflect the remarkable cellular heterogeneity of GSCs and their RNA diversity.

### ADAR1 promotes GSC proliferation and self-renewal.

**Figure 2:**
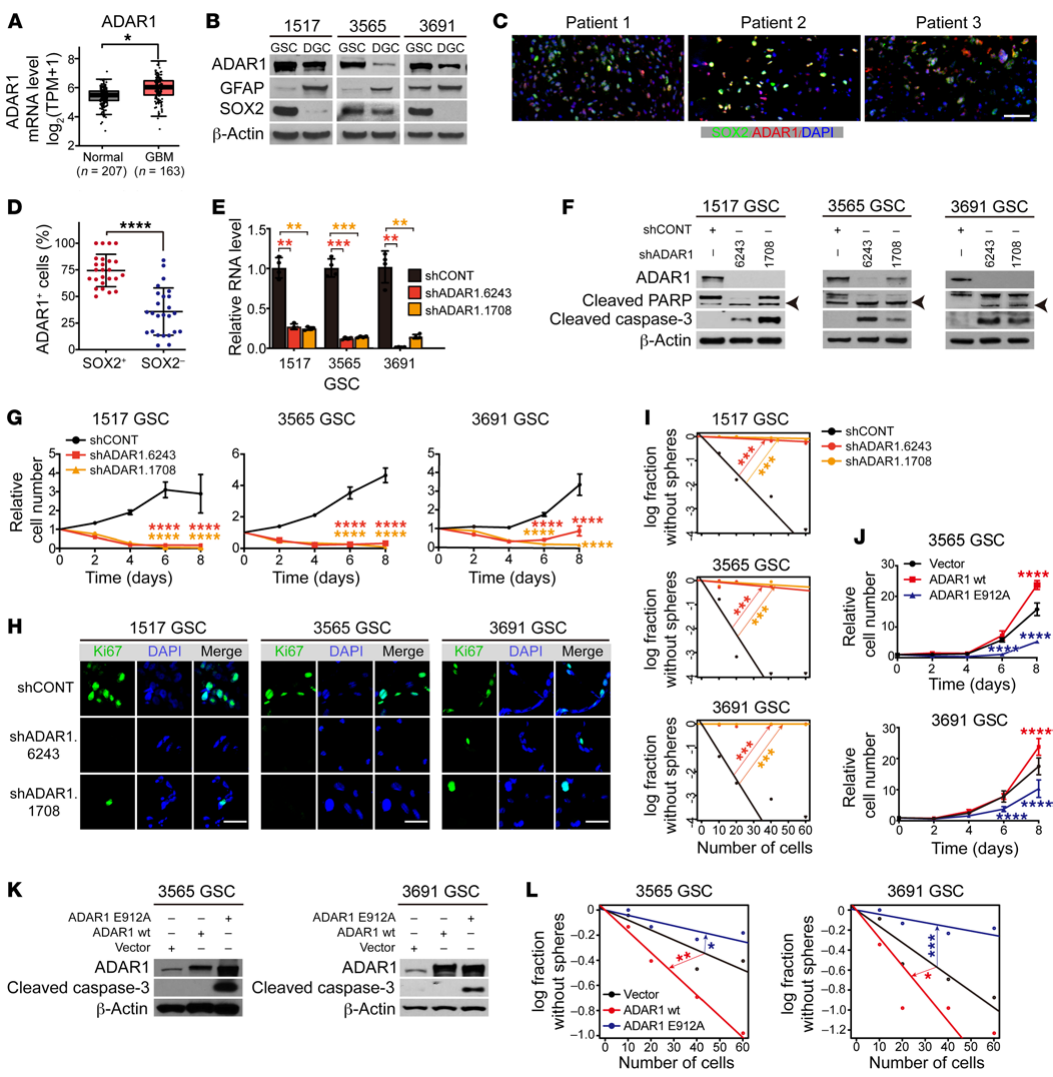
ADAR1 promotes GSC proliferation and self-renewal. See also Table 1 and Supplemental Figures 2 and 3. (**A**) ADAR1 expression levels in GBM (TCGA) and normal brain in the Genotype-Tissue Expression (GTEx) database. *P < 0.05. TPM, transcripts per million. (**B**) Immunoblotting of ADAR1 in paired GSCs and DGCs. GFAP and SOX2 serve as markers of differentiated and stem/progenitor cells. β-Actin served as loading control. (**C**) Immunofluorescence analysis of ADAR1 and SOX2. Scale bar: 50 μm. (**D**) Percentage of ADAR1^+^ cells among SOX2^+^ versus SOX2^–^ cells performed in **C**. Data were compared by Student’s *t* test and shown as mean ± SD. ****P < 0.0001. (**E**) ADAR1 expression of GSCs transduced with shCONT or shADAR1. *n =* 4. Data was determined by ANOVA and shown as mean ± SD. **P < 0.01, ***P < 0.001. (**F**) Immunoblotting for ADAR1, PARP, and cleaved caspase-3 in GSCs transduced with shCONT or shADAR1. β-Actin served as loading control. Arrowheads indicate cleaved PARP. (**G**) Proliferation of GSCs transduced with shCONT or shADAR1. Data was determined by 2-way ANOVA with Dunnett’s multiple-comparison testand shown as mean ± SD. *n* = 5. ****P < 0.0001. (**H**) Immunofluorescence analysis of Ki67 of GSCs transduced with shCONT or shADAR1. Scale bars: 20 μm. (**I**) Extreme limiting dilution analysis (ELDA) for sphere formation of GSCs transduced with shCONT or shADAR1. Data was determined by pairwise tests. *n =* 24. ***P < 0.001. (**J**) Proliferation of GSCs transduced with vector, ADAR1 wt, or ADAR1 E912A. Data was determined as in **G**. *n =* 5. ****P < 0.0001. (**K**) Immunoblotting for ADAR1 and cleaved caspase-3 in GSCs transduced as in **J**. β-Actin served as loading control. (**L**) ELDA for sphere formation of GSCs transduced as in **J**. *n =* 24. *P < 0.05, **P < 0.01, ***P < 0.001.

To determine which ADAR(s) is/are functionally relevant to GBM, we correlated the mRNA levels of individual ADAR genes with levels in normal brain and survival in GBM using 3 large patient databases: TCGA, the Chinese Glioma Genome Atlas (CGGA), and the Repository of Molecular Brain Neoplasia Data (REMBRANDT). ADAR1 mRNA was overexpressed in GBM relative to normal brain (Figure 2A). Both ADAR1 and ADARB1 were elevated at the protein level in GSCs compared with NSCs and nonmalignant cells derived from epilepsy surgical resection specimens (Supplemental Figure 2, A and B), but only ADAR1 was negatively associated with patient survival in all three GBM databases (Supplemental Figure 2, C and D). ADAR1 protein levels were higher in cultured GSCs compared with matched depleting GBM cells (DGCs) (Figure 2B), and ADAR1 was selectively expressed in cultured GSCs, but not NSCs, by immunofluorescence staining (Supplemental Figure 2E). Consistently, ADAR1 was detected in GBM surgical specimens marked by SOX2, a GSC marker (Figure 2, C and D). Furthermore, the expression of each of the stem cell markers SOX2, SOX4, and BMI1 in TCGA GBM data sets was positively correlated to ADAR1 mRNA levels, but negatively correlated with the expression of both ADARB1 and ADARB2 (Supplemental Figure 2F). Together, these data strongly suggest a key contribution of ADAR1 to GSCs.

Given the connections between ADAR1 and GSCs, we next determined the functional contributions of ADAR1 to GSC maintenance. Using 2 independent, non-overlapping shRNAs against ADAR1, both shADAR1s, but not nontargeting control shRNA, reduced ADAR1 mRNA (Figure 2E and Supplemental Figure 3A) and protein (Figure 2F and Supplemental Figure 3B) in patient-derived GSCs and DGCs. ADAR1 depletion induced apoptosis and loss of GSC proliferation (Figure 2, F and G), but had minimal to modest effects on matched DGCs (Supplemental Figure 3, A–C). To identify the antitumor mechanisms triggered by ADAR1 depletion, we performed RNA-Seq in GSCs upon ADAR1 knockdown, showing that ADAR1 downregulation impaired expression of genes involved in cancer proliferation, particularly those involved in cell cycle control and DNA replication (Supplemental Figure 3, D and E). As a consequence, the mitotic index measured by Ki67 was dramatically reduced (Figure 2H) and was associated with reduced GSC sphere formation, as assessed by in vitro limiting dilution assay, a surrogate of self-renewal (Figure 2I). To confirm that ADAR1 enzymatic activity was responsible for changes in GSC growth and self-renewal, we transduced 2 patient-derived GSCs with an empty vector control, a FLAG-tagged wild-type ADAR1 (ADAR1 wt), or a FLAG-tagged, enzymatically dead mutant ADAR1 that contains a point mutation in its catalytic site (ADAR1 E912A). GSCs transduced with ADAR1 wt displayed increased proliferation and self-renewal relative to vector control (Figure 2, J–L, and Table 1). In contrast, catalytically dead ADAR1 reduced proliferation, induced apoptosis, and reduced sphere formation relative to both ADAR1 wt and vector control, suggesting function as a dominant negative (Figure 2, J–L, and Table 1). Taken together, our data support an essential role for ADAR1 in GSC viability, proliferation, and self-renewal, likely mediated by the editing activity of ADAR1.

### ADAR1-induced A-to-I RNA editing regulates GM2A expression.

**Figure 3:**
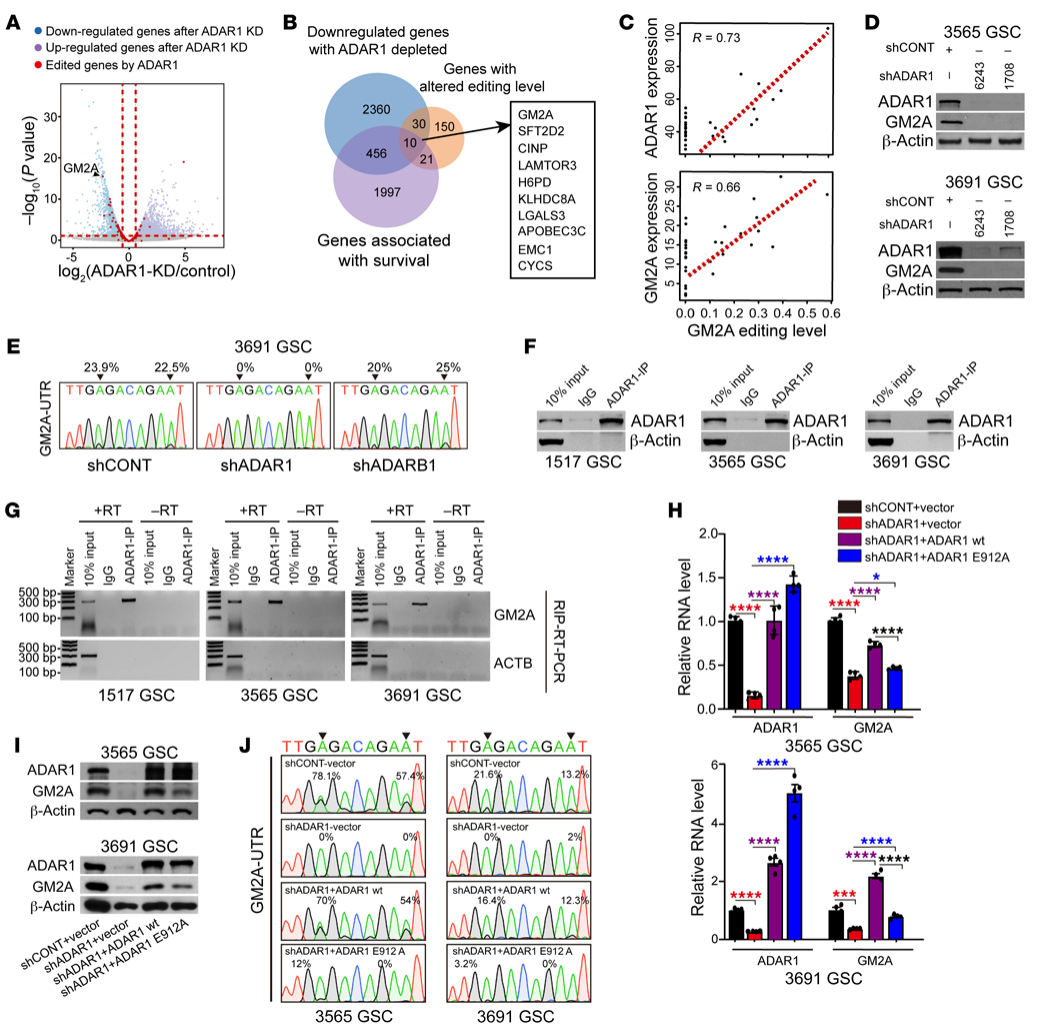
ADAR1-induced A-to-I RNA editing of GM2A regulates GM2A expression. See also Supplemental Figure 4. (**A**) Volcano plot of relative gene expression in GSCs transduced with shCONT or shADAR1. Purple and blue points designate upregulated and downregulated target genes, respectively, upon genetic targeting of ADAR1. Red points denote genes with editing sites. (**B**) Venn diagram of the overlapping genes.Blue circle: downregulated genes with ADAR1 depletion, purple circle: genes with negative prognostic significance in GBM in TCGA, orange circles: genes with alterations in editing levels. The rectangle shows 10 overlapping genes. (**C**) Top: Correlation between expression levels of ADAR1 and overall editing level of GM2A in GSCs. Bottom: Correlation between expression levels and overall editing levels of GM2A in GSCs. (**D**) Immunoblotting of ADAR1 and GM2A in GSCs transduced with shCONT or shADAR1. β-Actin served as loading control. (**E**) Sequence chromatograms of GM2A transcripts in 3691 GSCs transduced with shCONT, shADAR1, or shADARB1. Arrowheads indicate edited positions. Percentages indicate the calculated frequency of editing at selected positions. (**F**) Immunoblotting of ADAR1 IP in indicated GSCs. β-Actin served as a nonspecific control. (**G**) ADAR1 RNA immunoprecipitation (RIP) to pull down GM2A mRNA in indicated GSCs. ACTB served as nonspecific control. +RT and –RT: with and without RTase in reverse transcription. (**H**) ADAR1 and GM2A mRNA expression in indicated GSCs transduced with shCONT or shADAR1 with simultaneous transduction with vector control, wild-type ADAR1, or editing-dead E912A ADAR1 mutant. *n* = 4. Quantitative data from 4 independent experiments are shown as mean ± SD. Statistical significance was determined by ANOVA. *P < 0.05, ***P < 0.001, ****P < 0.0001. (**I**) Immunoblotting of ADAR1 and GM2A in indicated GSCs performed in **H**. β-Actin served as loading control. (**J**) Sequence chromatograms of the GM2A transcript performed in **I**. Arrowheads indicate edited positions.

To identify critical ADAR1 targets in GSCs, we performed RNA-Seq in ADAR1-knockdown GSCs and intersected the results with GSC-specific A-to-I editing events. We prioritized target genes that were (a) downregulated upon ADAR1 knockdown, (b) reduced in editing levels, and (c) negatively associated with GBM patient survival in the TCGA database. We identified 10 genes using these criteria (Figure 3, A and B), among which we further prioritized targets by comparing the editing levels of each gene with the expression of both ADAR1 and the corresponding gene. GM2A showed the highest expression in GSCs, the greatest reduction in mRNA upon ADAR1 knockdown, and the best correlation between expression and A-to-I editing levels (Figure 3, A and C, and Supplemental Figure 4, A and B).

GM2A is a small glycolipid transport protein, known as a GM2 ganglioside activator, that presents GM2 gangliosides to hexosaminidase (HEX) family members to promote ganglioside catabolism, which takes place predominantly in the central nervous system (48–50). Targeting ADAR1 expression in GSCs reduced protein and mRNA levels of both ADAR1 and GM2A (Figure 3D and Supplemental Figure 4C), supporting ADAR1 as an upstream regulator of GM2A. Next, we characterized the editing events in GM2A and identified 2 prevalent GSC-specific A-to-I edits in its 3′-UTR (Figure 3E). By cloning the reverse transcriptase PCR products followed by Sanger sequencing, we found that these RNA editing events were diminished in GSCs upon transduction with shADAR1, but not nontargeting control shRNA (shCONT) or shADARB1 (Figure 3E). Based on published ADAR1 CLIP-Seq data generated in U87MG glioma cells (51), ADAR1 bound in the 3′-UTR of GM2A (Supplemental Figure 4D). We confirmed such binding in GSCs by performing ADAR1 RNA immunoprecipitation–PCR (RIP-PCR) (Figure 3, F and G). These data demonstrate that ADAR1 directly acts on GM2A transcripts in GSCs.

To further demonstrate that ADAR1-mediated RNA editing is responsible for enhanced GM2A expression, we performed rescue experiments with either shRNA-resistant wild-type ADAR1 (ADAR1 wt) or a mutant ADAR1 that contains a point mutation in its catalytic site (ADAR1 E912A). While both ADAR1 wt and ADAR1 E912A were expressed at equivalent levels, only ADAR1 wt rescued GM2A expression and editing levels in shADAR1-transduced GSCs (Figure 3, H–J). Collectively, these data show that ADAR1-mediated RNA editing regulates GM2A expression in GSCs. We note that a smaller change in GM2A editing in 3691 GSCs gave more drastic effects on GM2A expression in comparison with 3565 GSCs, which likely reflects certain unknown variations among patient-derived GSCs that render some GSC isolates more sensitive to editing change than other isolates.

### GM2A contributes to GSC survival, proliferation, and self-renewal.

**Figure 4:**
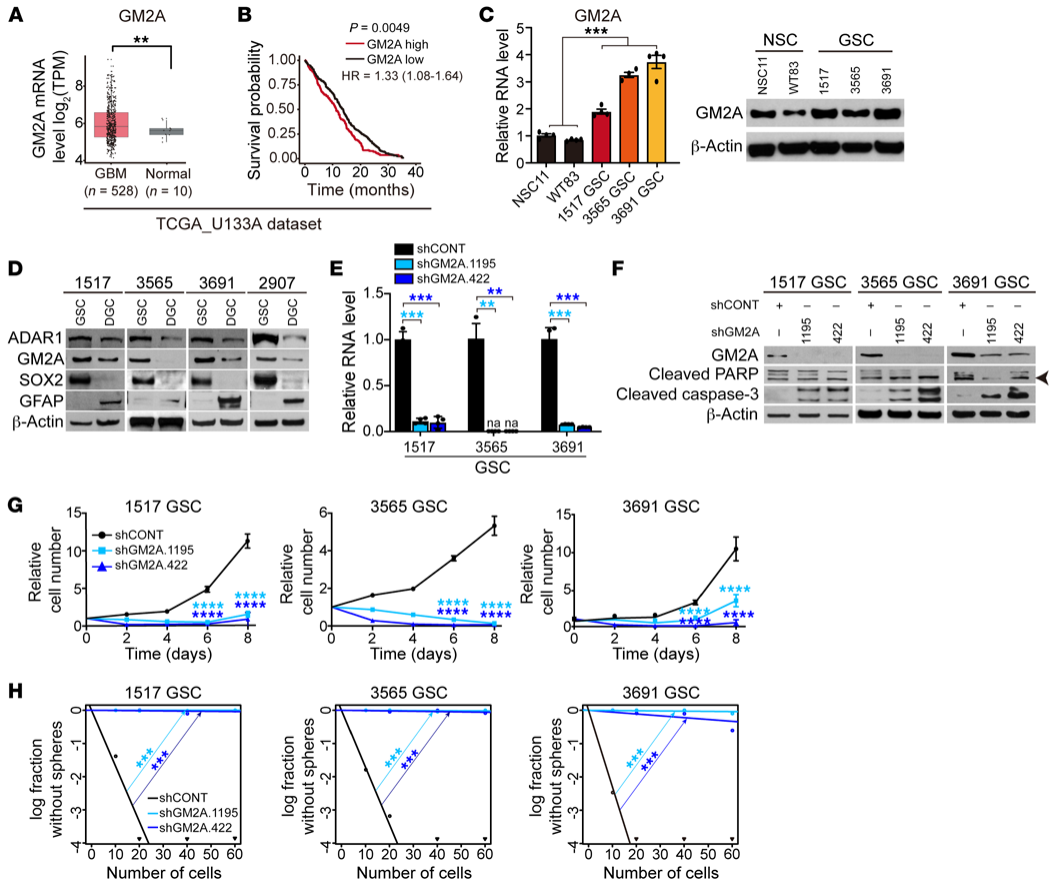
GM2A maintains GSC survival, proliferation, and self-renewal. See also Table 2. (**A**) Gene expression levels of GM2A in normal brain or gliomas in the TCGA database. Statistical significance was determined by Wilcoxon’s signed-rank test. **P < 0.01. (**B**) Kaplan-Meier survival curves of glioma patients with high or low GM2A expression in TCGA. *P* value was determined by log-rank test. (**C**) Left: Comparative GM2A mRNA expression in NSCs (NSC11 and WT83) and GSCs (1517, 3565, and 3691) by reverse transcriptase PCR. *n =* 4. Statistical significance was determined by ANOVA. ***P < 0.001. Right: Western blotting for GM2A in NSCs (NSC11 and WT83) and GSCs (1517, 3565, and 3691). β-Actin served as loading control. (**D**) Western blotting for ADAR1 and GM2A in matched GSCs and DGCs. GFAP and SOX2 served as markers of differentiated or stem/progenitor cells. β-Actin served as loading control. (**E**) GM2A mRNA expression in GSCs (1517, 3565, and 3691) transduced with shCONT or shGM2A. na, not available. *n =* 4. Quantitative data from 4 independent experiments are shown as mean ± SD (error bars). Statistical significance was determined by ANOVA. **P < 0.01, ***P < 0.001. (**F**) Western blotting for GM2A, PARP, and cleaved caspase-3 in GSCs (1517, 3565, and 3691) transduced with shCONT or shGM2A. β-Actin served as loading control. Arrowhead indicates cleaved PARP. (**G**) Proliferation of GSCs (1517, 3565, and 3691) transduced with shCONT or shGM2A as determined by CellTiter-Glo. Quantitative data from 5 technical experiments are shown as mean ± SD (error bars). *n =* 5. Statistical significance was determined by 2-way ANOVA with Dunnett’s multiple-comparison test. ****P < 0.0001. (**H**) ELDA for in vitro sphere formation of GSCs (1517, 3565, and 3691) transduced with shCONT or shGM2A. *n =* 24. Pairwise tests for differences in stem cell frequencies. ***P < 0.001.

GM2A was upregulated in GBM relative to normal brain and negatively associated with GBM patient survival in the TCGA GBM data set (Figure 4, A and B). GM2A expression was elevated at both the mRNA and protein levels in cultured GSCs compared with NSCs (Figure 4C). To determine GM2A expression within the GBM hierarchy, we compared GSCs with matched DGCs differentiated by serum exposure, revealing a consistent decrease in ADAR1 and GM2A expression in DGCs, which was coincident with diminished SOX2 expression (Figure 4D).

To determine whether loss of function of GM2A phenocopied the loss of ADAR1, we used 2 independent shRNAs to knock down GM2A and assessed the impact of GM2A depletion on GSCs (Figure 4E). Similarly to ADAR1 knockdown, silencing of GM2A in 3 patient-derived GSCs induced apoptosis, as indicated by strongly increased cleaved caspase-3 (without induction of cleaved PARPγ) (Figure 4F), retarded GSC growth (Figure 4G), and abolished sphere formation in limiting dilution assays compared with a nontargeting control shRNA (Figure 4H and Table 2). Together, these data demonstrate that GM2A is required to maintain the tumorigenic potential of GSCs, although other genes likely contribute to this process.

### GM2 ganglioside catabolism is critical to maintain GSC stemness.

**Figure 5:**
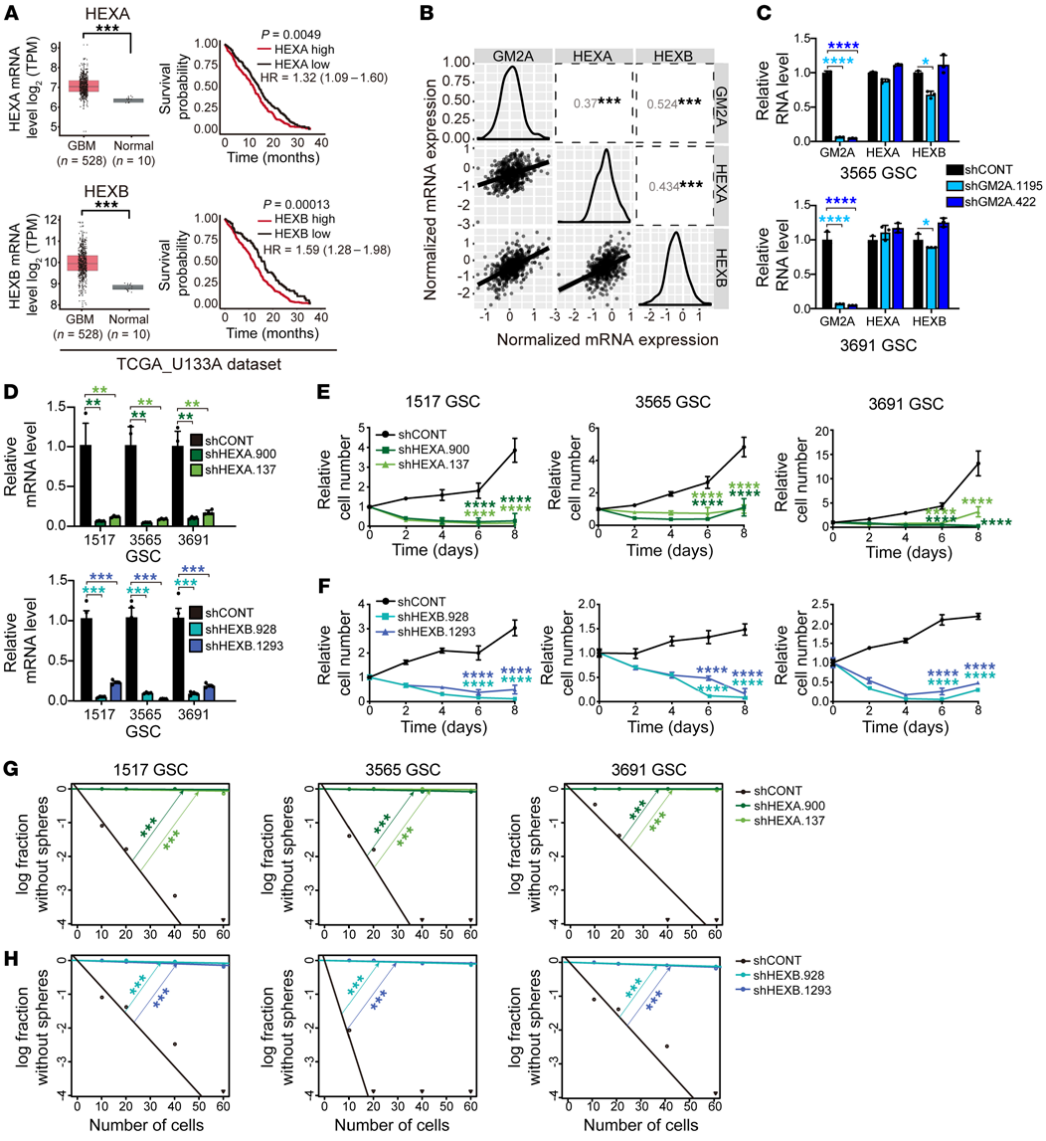
The GM2 ganglioside catabolic pathway maintains GSCs. (**A**) Left: Gene expression levels of HEXA and HEXB in normal brain and gliomas in TCGA. Statistical significance was determined by Wilcoxon’s signed-rank test. ***P < 0.01. Right: Kaplan-Meier survival curves of glioma patients with high or low HEXA or HEXB expression in TCGA. *P* value was determined by log-rank test. (**B**) Pairwise correlation analysis between GM2A, HEXA, and HEXB gene expression data from TCGA GBM data sets. Correlation coefficient (*R*) values are shown. (**C**) mRNA expression of HEXA and HEXB in GSCs (3565 and 3691) transduced with shCONT or shGM2A. Quantitative data from 3 independent experiments are shown as mean ± SD (error bars). *n =* 3. Statistical significance was determined by ANOVA. *P < 0.05, ****P < 0.0001. (**D**) HEXA (top) and HEXB (bottom) mRNA expression in GSCs (1517, 3565, and 3691) transduced with shCONT, shHEXA, or shHEXB. *n =* 4. Quantitative data from 4 independent experiments are shown as mean ± SD (error bars). Statistical significance was determined by ANOVA. **P < 0.01, ***P < 0.001. (**E**) Proliferation of GSCs (1517, 3565, and 3691) transduced with shCONT or shHEXA determined by CellTiter-Glo. *n =* 5. Quantitative data from 5 technical experiments are shown as mean ± SD (error bars). Statistical significance was determined by 2-way ANOVA with Dunnett’s multiple-comparison test. ****P < 0.0001. (**F**) Proliferation of GSCs (1517, 3565, and 3691) transduced with shCONT or shHEXB determined by CellTiter-Glo. *n =* 5. Quantitative data from 5 technical experiments are shown as mean ± SD (error bars). Statistical significance was determined by 2-way ANOVA with Dunnett’s multiple-comparison test. ****P < 0.0001. (**G**) ELDA for in vitro sphere formation of GSCs (1517, 3565, and 3691) transduced with shCONT or shHEXA. *n =* 24. Pairwise tests for differences in stem cell frequencies. ***P < 0.001. (**H**) Analyses identical to those in **G** were performed for HEXB.

GM2A is a sphingolipid protein that presents GM2 gangliosides to the lysosomal β-hexosaminidase enzymes (HEXA and HEXB) for catabolism in lysosomes (48, 52). Like GM2A, these GM2 ganglioside catabolism pathway genes were upregulated in GBM relative to normal brain, and both HEXA and HEXB were negatively associated with GBM patient prognosis (Figure 5A). GM2A expression correlated with HEXA and HEXB expression in GBM (Figure 5B), which is consistent with their coordinated function. However, as expected, GM2A did not directly regulate the expression of either HEXA or HEXB, as targeting GM2A expression did not significantly alter the RNA levels of either HEXA or HEXB (Figure 5C). To determine the roles of HEXA and HEXB in GSCs, we targeted HEXA and HEXB separately, each with 2 independent shRNAs (Figure 5D). Similarly to GM2A depletion, loss of either HEXA or HEXB decreased GSC growth (Figure 5, E and F) and abolished tumor sphere formation (Figure 5, G and H). These results suggest that elevated GM2 ganglioside catabolism is critical to maintain GSC stemness.

**Figure 6:**
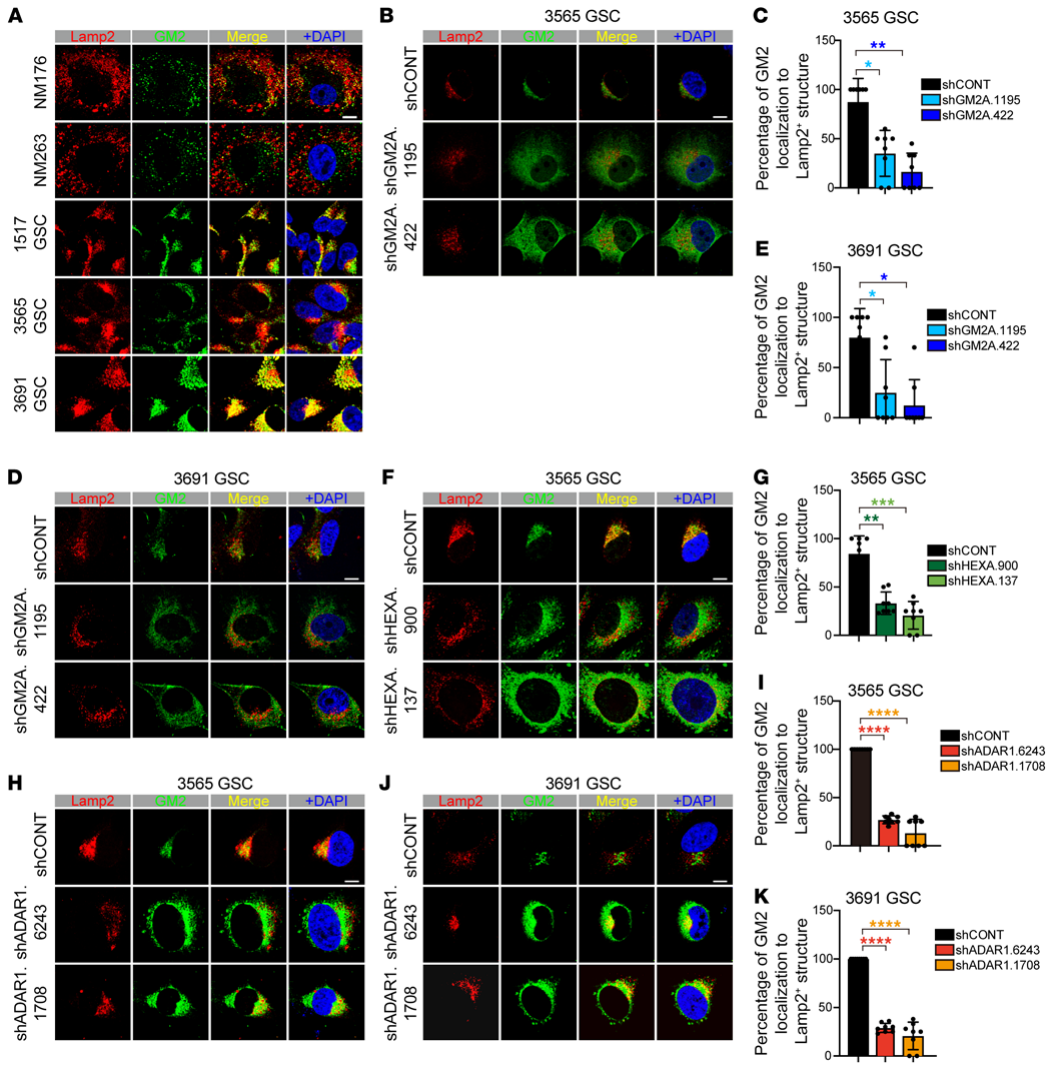
Disruption of GM2 ganglioside catabolism relocates GM2 gangliosides. (**A**) Immunofluorescence analysis of nonmalignant cells derived from epilepsy surgical specimens (NM176 and NM263) and GSCs (1517, 3565, and 3691) with lysosome marker Lamp2 and GM2 ganglioside. DAPI indicates nuclei. Scale bar: 10 μm. (**B**) Immunofluorescence analysis of 3565 GSCs transduced with shCONT or shGM2A with lysosome marker Lamp2 and GM2 ganglioside. *n =* 2 biological replicates. DAPI indicates nuclei. Scale bar: 10 μm. (**C**) Statistical values for experiments performed in **B**. Data are shown as mean ± SD. Statistical significance was compared by Student’s *t* test. *P < 0.05, **P < 0.01. (**D**) Immunofluorescence analysis of 3691 GSCs transduced with shCONT or shGM2A with lysosome marker Lamp2 and GM2 ganglioside. *n =* 2 biological replicates. DAPI indicates nuclei. Scale bar: 10 μm. (**E**) Statistical values for experiments performed in **D**. Data are shown as mean ± SD. Statistical significance was compared by Student’s *t* test. *P < 0.05. (**F**) Immunofluorescence analysis of 3565 GSCs transduced with shCONT or shHEXA with lysosome marker Lamp2 and GM2 ganglioside. *n =* 2 biological replicates. DAPI indicates nuclei. Scale bar: 10 μm. (**G**) Statistical values for experiments performed in **F**. Data are shown as mean ± SD. Statistical significance was compared by Student’s *t* test. **P < 0.01, ***P < 0.001. (**H**) Immunofluorescence analysis of 3565 GSCs transduced with shCONT or shADAR1 with lysosome marker Lamp2 and GM2 ganglioside. *n =* 2 biological replicates. DAPI indicates nuclei. Scale bar: 10 μm. (**I**) Statistical values for experiments performed in **H**. Data are shown as mean ± SD. Statistical significance was compared by Student’s *t* test. ****P < 0.0001. (**J**) Immunofluorescence analysis of 3691 GSCs transduced with shCONT or shADAR1 with lysosome marker Lamp2 and GM2 ganglioside. *n =* 2 biological replicates. DAPI indicates nuclei. Scale bar: 10 μm. (**K**) Statistical values for experiments performed in **J**. Data are shown as mean ± SD. Statistical significance was compared by Student’s *t* test. ****P < 0.0001.

Given the functional contributions of GM2A, HEXA, and HEXB to lysosomal degradation of GM2 gangliosides, we next investigated lysosomal localization of GM2 gangliosides in GSCs in comparison with nonmalignant neural cultures from epilepsy patient surgical specimens. In GSCs, GM2 gangliosides strongly colocalized with lysosomes, as indicated by coimmune staining between the lysosomal marker Lamp2 and GM2 gangliosides (Figure 6A). In contrast, in nonmalignant cultures, GM2 gangliosides were located proximal to, but not in, lysosomes (Figure 6A). Upon shRNA targeting of GM2A, lysosomes appeared morphologically intact and remained near nuclei, while GM2 gangliosides became dispersed into the cytoplasm with only partial lysosomal localization in GSCs (Figure 6, B–E). Likewise, shRNA-mediated depletion of HEXA in GSCs induced a similar phenotype as GM2A depletion with a disrupted localization GM2 ganglioside to the lysosome (Figure 6, F and G). Finally, targeting ADAR1 in GSCs through shRNA transduction induced a similar loss of lysosomal localization of GM2 gangliosides (Figure 6, H–K). Collectively, these results support similar roles of ADAR1, GM2A, and HEXA in GM2 ganglioside targeting to lysosomes and GSC maintenance.

### Pharmacologic inhibition of GSC self-renewal by exploiting of the ADAR1/GM2 axis.

**Figure 7:**
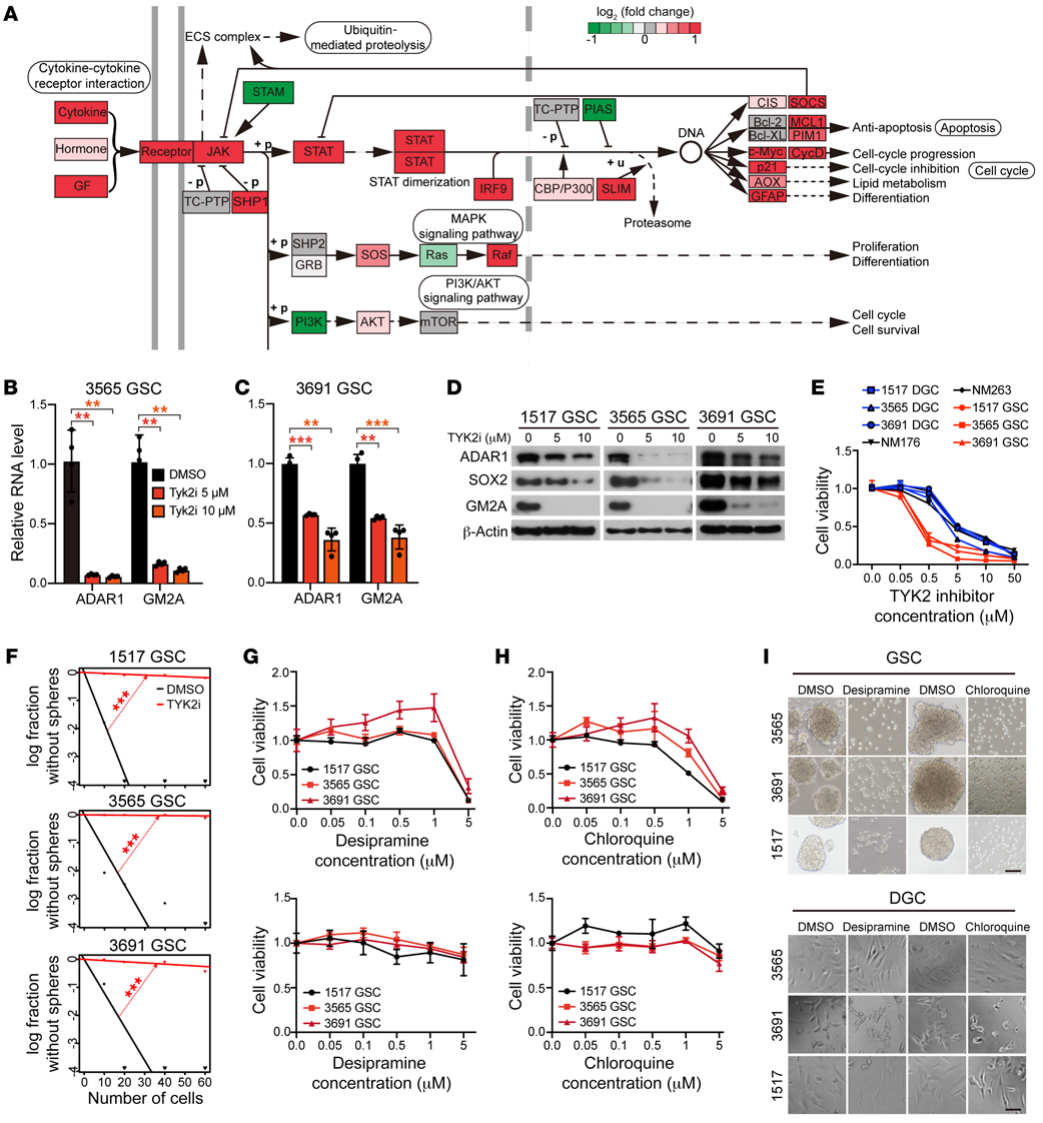
Pharmacologic targeting of GSC self-renewal through the ADAR1/GM2 axis. See also Supplemental Figure 5. (**A**) Gene set enrichment analysis based on KEGG pathway annotation of JAK/STAT signaling pathway genes in GBM informed by TCGA data. (**B**) ADAR1 and GM2A mRNA expression in 3565 GSCs treated with vehicle control (DMSO) or different concentrations of a TYK2 inhibitor. *n =* 4. Quantitative data from 4 independent experiments are shown as mean ± SD (error bars). Statistical significance was determined by ANOVA. **P < 0.01. (**C**) ADAR1 and GM2A mRNA expression in 3691 GSCs treated with vehicle control (DMSO) or several concentrations of a TYK2 inhibitor. *n =* 4. Quantitative data from 4 independent experiments are shown as mean ± SD (error bars). Statistical significance was determined by ANOVA. **P < 0.01, ***P < 0.001. (**D**) Western blotting for ADAR1, SOX2, and GM2A in GSCs (1517, 3565, and 3691) treated with vehicle control (DMSO) or TYK2 inhibitor. (**E**) Comparative concentration response curves for GSCs (1517, 3565, and 3691), matched DGCs (1517, 3565, and 3691), and nonmalignant cells derived from epilepsy surgical specimens (NM176 and NM263) treated with increasing concentrations of a TYK2 inhibitor for 2 days. (**F**) ELDA for in vitro sphere formation of GSCs (1517, 3565, and 3691) treated with vehicle control (DMSO) or a TYK2 inhibitor. *n =* 24. Pairwise tests for differences in stem cell frequencies. ***P < 0.001. (**G**) Cell viability of GSCs (1517, 3565, and 3691; top) and matched DGCs (1517, 3565, and 3691; bottom) with increasing concentrations of desipramine. (**H**) Cell viability of GSCs (1517, 3565, and 3691; top) and matched DGCs (1517, 3565, and 3691; bottom) with increasing concentrations of chloroquine. (**I**) Sphere formation of GSCs (1517, 3565, and 3691; top) and matched DGCs (1517, 3565, and 3691; bottom) with DMSO, desipramine, or chloroquine. Scale bars: 50 μm.

Having established the importance of the ADAR1/GM2 axis in maintaining GSC self-renewal and stemness, we next exploited strategies to intervene with this pathway. As ADAR1 is regulated by interferon through the JAK/STAT pathway (53, 54), we performed gene set enrichment analysis based on Kyoto Encyclopedia of Genes and Genomes (KEGG) pathways in the GBM TCGA data set, revealing that the JAK/STAT signaling pathway was indeed one of the most differentially expressed gene sets in GBMs (Figure 7A). Several genes in the JAK/STAT pathway correlated with ADAR1 expression in GBM (Supplemental Figure 5A). Although the molecular link between ADAR1 expression and the JAK pathways remains to be fully elucidated in future studies, we elected to first explore the therapeutic potential of such connection by treating GSCs with selective inhibitors against 4 JAK family members: JAK1, JAK2, JAK3, and TYK2. We found that while inhibitors of JAK1, JAK2, and JAK3 showed minimal impact on ADAR1 expression (Supplemental Figure 5, B–D), a specific TYK2 inhibitor greatly diminished ADAR1 mRNA (Figure 7, B and C) and protein (Figure 7D) levels in GSCs. In line with ADAR1 as an upstream regulator, GM2A expression progressively diminished in a concentration-dependent manner (Figure 7D).

We next utilized the TYK2 inhibitor to determine its potency in selective inhibition of GSC proliferation relative to either nonmalignant cells or matched DGCs under the same concentrations (Figure 7E). TYK2 inhibitor treatment also reduced GSC self-renewal (Figure 7F). Given our finding of a link between ADAR1-mediated RNA editing and GM2 ganglioside catabolism, we explored the translational potential of targeting GM2. Certain cationic amphiphilic drugs, including desipramine and chloroquine, induce phospholipidosis and inhibit GM2 hydrolysis (55). Treatment with either desipramine or chloroquine reduced GSC proliferation (Figure 7, G and H, top panels) and sphere formation (Figure 7I, top panel). In contrast and aligned with GSC-specific effects, desipramine or chloroquine minimally affected the of DGC proliferation (Figure 7, G and H, bottom panels) and sphere formation (Figure 7I, bottom panel).

### ADAR1 and GM2A promote in vivo tumorigenesis.

**Figure 8:**
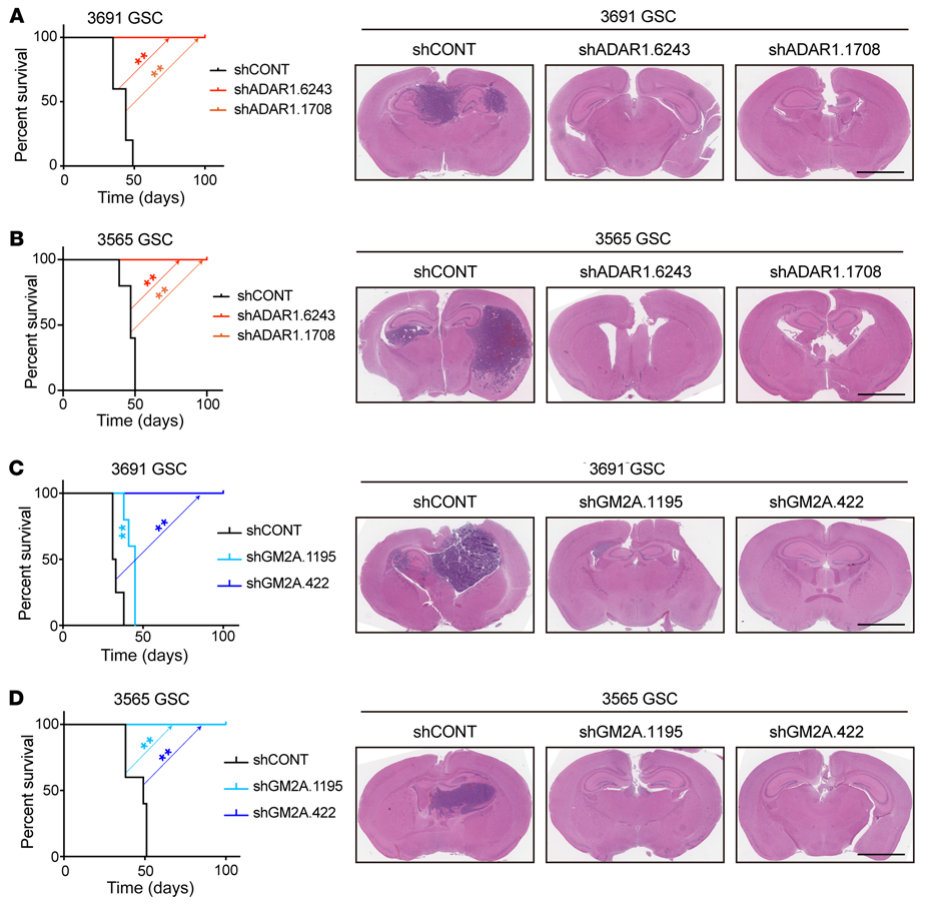
Targeting ADAR1 or GM2A attenuates in vivo tumor growth. (**A**) Survival analysis of NSG mice bearing intracranially implanted patient-derived 3691 GSCs transduced with shCONT or 1 of 2 non-overlapping shADAR1s (left panel). Statistical significance was determined by log-rank (Mantel-Cox) test. GSCs were implanted intracranially into NSG mice, and tumor formation was determined by H&E staining (right panel). Scale bar: 2 mm. *n =* 5 per group, 2 biological replicates. (**B**) Survival analysis of NSG mice bearing intracranially implanted patient-derived 3565 GSCs transduced with shCONT or 1 of 2 non-overlapping shADAR1s (left panel). Statistical significance was determined by log-rank (Mantel-Cox) test. GSCs were implanted intracranially into NSG mice, and tumor formation was determined by H&E staining (right panel). Scale bar: 2 mm. *n =* 5 per group, 2 biological replicates. (**C**) Survival analysis of NSG mice bearing intracranially implanted patient-derived 3691 GSCs transduced with shCONT or 1 of 2 non-overlapping shGM2As (left panel). Statistical significance was determined by log-rank (Mantel-Cox) test. GSCs were implanted intracranially into NSG mice, and tumor formation was determined by H&E staining (right panel). Scale bar: 2 mm. *n =* 5 per group. (**D**) Survival analysis of NSG mice bearing intracranially implanted patient-derived 3565 GSCs transduced with shCONT or 1 of 2 non-overlapping shGM2As (left panel). Statistical significance was determined by log-rank (Mantel-Cox) test. GSCs were implanted intracranially into NSG mice, and tumor formation was determined by H&E staining (right panel). Scale bar: 2 mm. *n =* 5 per group. **P < 0.01.

As in vivo tumor formation is an essential feature of GSCs, we interrogated ADAR1 and GM2A dependencies in proof-of-principle tumor xenograft experiments. GSCs transduced with 1 of 2 independent shADAR1s or a control shRNA encoding a nontargeting sequence were transplanted into the brains of immunocompromised mice. Mice bearing intracranial GSCs transduced with shADAR1 demonstrated eradication of tumor formation relative to mice bearing GSCs expressing the shRNA control (Figure 8, A and B). We further extended the analysis by examining the GM2A dependency. Consistent with a critical role of GM2A in GSC maintenance, mice with transplanted GSCs expressing shGM2A displayed longer survival and reduced tumor volume compared with those transduced with the control shRNA (Figure 8, C and D). Collectively, these results strongly suggest that therapeutic targeting of the TYK2/ADAR1/GM2A axis may serve as a new clinical strategy for GBM treatment.

## Discussion

A-to-I RNA editing is an important post-transcriptional mechanism to regulate RNA metabolism. The biological role of RNA editing has been explored in development and autoimmune diseases, but more recent studies point to vital contributions to cancer. The advent of RNA-Seq has greatly accelerated the recognition of the function of RNA editing in both physiological and disease contexts (42, 43, 56, 57), including cancer development and progression (16, 57, 58). Complicating the connection between RNA editing and cancer, A-to-I RNA editing enzymes can serve as either tumor suppressors or oncogenes. ADAR1 has been suggested to maintain both normal and cancer stem cells through multiple mechanisms (59–64). Shared A-to-I RNA editing sites specific to a large panel of GSCs were relatively restricted, but the genes most specifically edited in GSCs included genes functionally associated with GSC maintenance, such as *PTPRZ1* (65, 66), *WWTR1* (67), and *CPT1A* (68).

Despite the fact that GBM was among the first cancer types studied by TCGA and other large omics efforts, precision medicine has shown limited benefit in managing GBM. In our current study, we characterized the landscapes of A-to-I RNA editing in GSCs and established vital contribution of elevated ADAR1 to GBM stemness. As altered A-to-I RNA editing events have been found in multiple cancers, RNA editing may contribute to tumorigenesis by globally modulating the dsRNA sensing system or via specific target transcripts, as suggested by recent literature (36, 69). Our data suggest that most RNA editing events are cancer specific, implying that altered RNA editing may use different mechanisms to enhance tumorigenesis in different tumor contexts. In the case of GBM, specific RNA editing events negatively correlated with GBM patient survival, which enabled us to prioritize key ADAR1-regulated editing events in GBM. As ADAR1 (and ADARB1) are enzymes, future therapeutics may be developed based on modulation of these activities. One challenge to this effort is that, like many molecular modifications, the effects of the ADARs on A-to-I editing are context specific with reported oncogenic and tumor-suppressive effects. For example, the reported tumor-suppressive effects of ADAR2 (ADARB1) on glioma (27, 38) may contrast with our findings as a result of the differential binding of ADAR1 and ADARB1 on specific RNAs and modification sites on RNAs or effects on the larger tumor compartment. Deletions of the non-enzymatic ADAR, ADAR3, have also been reported in gliomas (70), suggesting that the ADARs and specific RNA editing events may play context-specific roles. While GSCs represent a small fraction of tumor cells in GBM, targeting of these populations has repeatedly shown sustained antitumor effects on bulk tumors. Collectively, the role of A-to-I RNA editing and activities of specific ADARs bear sustained study as these pathways become potentially modified by pharmacologic agents.

One opportunity to address the molecular targeting of ADAR1 function is to identify potential upstream and downstream regulators. Through a sequential prioritization process, we discovered GM2A as an RNA editing target, as ADAR1 directly binds in the 3′-UTR of GM2A that carries an Alu sequence and catalyzes A-to-I RNA editing within the inverted repeat. Such ADAR1-mediated editing is linked to enhanced GM2A expression at both RNA and protein levels, which may be related to a gain of function in the transcript, as elucidated for other transcripts earlier (36, 71). While detailed regulatory mechanisms remain to be understood, the ADAR1/GM2 axis that links GSC self-renewal to GM2 ganglioside catabolism may inform future therapeutic paradigms.

GM2 gangliosides are glycans. Several functions of glycans have been implicated in cancers (72, 73), and GM2 promotes tumor cell invasion and migration (74–76). GM2 accumulates in lysosomes as a functional consequence of specific mutations in GM2A or its partners in GM2 degradation, HEXA and HEXB (77, 78). Mutations in these GM2 molecular regulators lead to inborn-error diseases, such as AB variant and Tay-Sachs disease (77). We now demonstrate that GM2A depletion inhibits GSC growth and self-renewal, with associated changes in both GM2 levels and distribution in the cytoplasm. Direct GM2A inhibitors have not been developed, but several cationic amphiphilic drugs, including desipramine and chloroquine, induce phospholipidosis and inhibit GM2 hydrolysis (55), suggesting that these drugs may be repurposed for GBM therapy.

In orthogonal studies, we sought regulatory mechanisms upstream of ADAR1 and identified a TYK2 inhibitor as a potential therapeutic agent. TYK2 is a nonreceptor tyrosine protein kinase that associates with the cytoplasmic domains of type I and type II cytokine receptors to transduce cytokine signaling by phosphorylating receptor subunits (79). As a member of the JAK family and a component of the type I and type III IFN signaling pathways, inhibition of TYK2 modulates ADAR1 expression to block GSC proliferation and self-renewal, which may indicate that ADAR1 is one of the targets in GSCs, thus supporting its potential clinical utility.

The JAK/STAT family member STAT3 maintains GSCs (80, 81) and itself undergoes intronic A-to-I RNA editing to regulate its expression by alternative splicing in the MCF7 breast cancer cell line (82). In addition, GM2 marks stem-like pancreatic cancer cells and may be regulated by STAT3 (83). We did not prioritize STAT3 for further investigation in our studies, as TYK2 appeared most promising, but we note that in the TCGA data set ADAR1 expression correlated with STAT3 (*R* = 0.32; Supplemental Figure 5A), less significantly than with TYK2 (*R* = 0.66). Further, STAT3 transcripts, even in the previously mentioned intron, did not undergo editing in our GSCs (data not shown). Thus, STAT3 regulation of GSCs appears less likely to be connected to ADAR1 function or A-to-I editing.

Notably, each node in the TYK2/ADAR1/GM2A axis has been associated with immune regulation. For example, in melanoma cells, loss of ADAR1 increases sensing of IFN-induced dsRNA to overcome the resistance to the immune checkpoint blockade (69, 84). In fact, we find that multiple IFN pathway genes are highly edited, suggesting that elevated RNA editing may impact both the dsRNA sensing system and specific tumor-promoting genes. This might contribute to the well-known property of GBM as an immunologically cold tumor and the fact that most immune therapies show modest, if any, therapeutic benefit. In future studies, we expect to leverage these findings to target the TYK2/ADAR1/GM2A axis to enhance immunotherapy in GBM treatment.

In conclusion, we present a global map of A-to-I editing events that occur in GSCs from which we identify specific events that contribute to GSC self-renewal. By pursuing one of the prevalent edited genes, *GM2A*, we uncover an unprecedented link of regulated catabolism to GSC stemness, and we provide a series of proof-of-concept experiments for exploiting the TYK2/ADAR1/GM2A axis as a potential strategy for GBM treatment.

## Methods

### Tumor dissociation, derivation, and culture of glioma stem cells, nonmalignant brain cultures, and neural stem cells.

All GBM cells were derived from the primary patient tumors or xenografted tumors. The dissociation procedure was performed as previously described (5) according to the manufacturer’s instructions. All patient studies were conducted in accordance with the Declaration of Helsinki. 1517, 3565, 3691, and 1919 GSCs were derived by our laboratory and transferred via materials transfer agreement from Duke University. Nonmalignant brain cultures (NM176 and NM263) were derived from human epilepsy resection specimens. The human NSC lines NSC11 and WT83 (ALSTEM) were derived from human induced pluripotent stem cells. All GSCs and NSCs were cultured in vitro in neurobasal medium supplemented with B27 (Invitrogen), l-glutamine, sodium pyruvate, 1% penicillin/streptomycin, 20 ng/mL basic fibroblast growth factor, and 20 ng/mL epidermal growth factor for at least 6 hours to recover expression of surface antigens. Both GSCs and differentiated GBM cells (DGCs) were collected using prospective sorting followed by assays to confirm stem cell marker expression, sphere formation, and secondary tumor initiation. Matched GSC and DGC experiments were performed. DGCs were maintained in DMEM supplemented with 10% FBS (Gibco) to maintain differentiation. Each tumor model was sent to short tandem repeat analyses before use. Mycoplasma testing was performed by quantitative PCR cellular supernatants at least every 6 months. All cells were thawed within 1 month of these experimental procedures.

### Proliferation and sphere formation assays.

Cell proliferation experiments were conducted by plating of cells of interest at a density of 1500 cells per well in a 96-well plate with 5 replicates. CellTiter-Glo (Promega) was used to measure cell proliferation. All data were normalized to day 0 and presented as mean ± SD. Sphere formation was measured by in vitro limiting dilution, as previously reported (85). Briefly, decreasing numbers of cells per well (60, 40, 20, and 10) were plated into 96-well plates. The presence and number of spheres in each well were recorded 10 days after plating. Extreme limiting dilution analysis was performed using software available at http://bioinf.wehi.edu.au/software/elda, as previously described (85). All tumor sphere and proliferation experiments were performed 3 times.

### Western blotting.

Cells were collected and lysed in RIPA buffer (50 mM Tris-HCl pH 7.5, 150 mM NaCl, 0.5% NP-40, and 50 mM NaF with protease inhibitors), then incubated on ice for 15 minutes. Lysates were centrifuged at 4°C for 10 minutes at 12,000 rpm, and supernatants were collected. The Bradford assay (Bio-Rad Laboratories) was used for determination of protein concentrations. Equal amounts of protein samples were mixed with 2× SDS Laemmli loading buffer, boiled for 10 minutes, and electrophoresed using SDS-PAGE, then transferred onto PVDF membranes. TBST supplemented with 5% nonfat dry milk was used for blocking for 1 hour followed by blotting with primary antibodies at 4°C overnight. Blots were washed 4 times for 5 minutes with TBST and then incubated with appropriate secondary antibodies for 1 hour. The blots were developed by SuperSignal West Pico PLUS Chemiluminescent Substrate (Thermo Fisher Scientific) and Autoradiography Film (Denville Scientific Inc). All the antibodies are listed in Supplemental Table 1. See complete unedited blots in the supplemental material.

### Immunofluorescence.

Tumor samples from GBM patients were fixed in 4% paraformaldehyde overnight at 4°C, followed by overnight cryoprotection with 20% sucrose in PBS at 4°C. Samples were then sectioned at a thickness of 7 μm. Sections were blocked with 1% BSA for 1 hour and then stained with anti-ADAR1 or anti-SOX2 antibodies at 4°C overnight. Sections were washed 4 times for 5 minutes and incubated with appropriate Alexa Fluor–conjugated secondary antibodies for 1 hour at 4°C followed by washing 3 times and counterstained with DAPI.

Cells were plated onto coverslips and grown to 50%–80% confluence in an incubator. Fixed cells were treated with 0.3% Triton X-100 for 10 minutes and then incubated with 1% BSA for 1 hour. Then the coverslips were incubated with a primary antibody at 4°C overnight. Cells were washed 4 times for 5 minutes and then incubated with appropriate Alexa Fluor–conjugated secondary antibodies for 1 hour at 4°C followed by washing 3 times and counterstained with DAPI. All microscopy images were obtained using a Leica SPE confocal microscope and processed in Adobe Photoshop CS6. Antibodies are listed in Supplemental Table 1.

### RNA isolation and quantitative reverse transcriptase PCR.

Trizol reagent (Sigma-Aldrich) was used to isolate total cellular RNA from cell pellets. The qScript cDNA Synthesis Kit (Quanta BioSciences) was used for reverse transcription into cDNA. Quantitative real-time PCR was performed with an Applied Biosystems 7900HT cycler using SYBRGreen PCR Master Mix (Thermo Fisher Scientific). All the primers are listed in Supplemental Table 2.

### RNA immunoprecipitation–quantitative PCR.

For ADAR1 immunoprecipitation, 2 μg of antibody was coupled to Dynabeads at 4°C for 2 hours in 200 μL lysis buffer (50 mM Tris-HCl pH 8.0, 150 mM NaCl, 2 mM EDTA, 1% NP-40, and proteinase inhibitor cocktail), and after washing with lysis buffer, cell extracts were added. The mix was incubated while being rotated at 4°C for 2 hours. After incubation, the beads were washed 4 times and separated into 2 parts. One part was for RNA extraction and reverse transcription into cDNA using the qScript cDNA Synthesis Kit (Quanta BioSciences); the other part was eluted with lysis buffer and analyzed by SDS-PAGE.

### Plasmids and lentiviral transduction.

All shRNAs used were purchased from Sigma-Aldrich and are listed in Supplemental Table 2. 293FT cells were used to generate lentiviral particles through cotransfection of the packaging vectors pCMV-dR8.2 and VSVG. Twelve hours after transfection, media were changed to neurobasal complete medium. Media containing lentiviral particles were collected by filtering with a 0.45 μm filter and concentrated with a Lenti-X concentrator (Takara), aliquoted to small vials, and stored at –80°C.

### In vivo tumorigenesis.

Healthy 4- to 6-week-old NSG mice (NOD.Cg-Prkdcscid Il2rgtm1Wjl/SzJ, The Jackson Laboratory) were randomly selected and used in this study for intracranial implantation. Intracranial xenografts were generated by implantation of human-derived GSCs into the right cerebral cortex of mice at a depth of 3.5 mm. Animals were monitored until neurological signs (such as hunched posture, gait changes, lethargy, and weight loss) were observed, and then they were sacrificed. Brains were harvested and fixed in 4% formaldehyde for at least 48 hours, stored in 70% ethanol at 4°C, and then cryosectioned. H&E staining was performed on sections for histological analysis. In parallel survival experiments, mice were observed until the development of neurological signs.

### Characterization of RNA variants and A-to-I RNA editing profiles.

After obtaining raw RNA-Seq sequencing reads for 31 GSC and 5 NSC samples, we first filtered the low-quality reads and cut adapters using Trimmomatic (86) with parameters SLIDINGWINDOW:2:20 MINLEN:38. RNA variations were called following the Best Practices recommendations for calling variants on the RNA-Seq data pipeline from the Genome Analysis Toolkit (GATK) (87). We required variants to be supported by at least 1 mismatched read with a base quality score ≥25, a mapping quality score ≥20, and coverage of each site ≥10. To remove DNA variations, we identified SNPs based on the corresponding whole exome sequencing (WES) libraries using GATK. All variations found by the exome sequencing data were filtered. We also removed all known DNA variations in the 1000 Genomes Project, dbSNP (database version 150; http://www.ncbi.nlm.nih.gov/SNP/), the University of Washington Exome Sequencing Project (http://evs.gs.washington.edu/EVS/), the Catalogue of Somatic Mutations in Cancer (COSMIC) database (88), and the ClinVar database (89). ANNOVAR (90) was used to re-annotate the remaining variations. To further remove false positive results, only variations curated in the RADAR database (91) were considered as editing sites. To ensure adequate statistical power, we identified the informative RNA editing sites among the detected RNA editing sites by requiring at least 10 samples, including normal samples. Finally, we obtained 6541 high-confidence RNA editing sites.

For a specific editing site, the editing level in a given sample was calculated as the number of edited reads divided by the total number of reads covering the site. We determined the overall gene editing level as the total number of reads at all known editing positions as compared with all reads covering the position (i.e., containing A and G nucleotides at the editing position). The sample overall editing levels were similar to the gene overall editing levels, which used the reads supporting all editing sites in each sample divided by the whole reads mapped to the editing sites. GSC-enriched editing sites were defined as editing sites for which the mean editing levels were higher in GSC samples compared with NSC samples. Eighty-six genes were defined stringently as GSC-specifically edited if (GSC mean editing level)/(GSC mean editing level + NSC mean editing level) was ≥0.75.

### RNA-Seq analysis.

All original RNA-Seq data on ADAR1 knockdown from GSC samples were deposited in the NCBI’s Gene Expression Omnibus (GEO) database (GEO GSE153270). Quality control was performed using Trimmomatic with parameters MINLEN: 38 and SLIDINGWINDOW: 2:20. Filtered reads were mapped to the reference human genome (hg38) using STAR (92) with the following parameters: --outFilterScoreMinOverLread 0.1, --outFilterMatchNminOverLread 0.1, --alignIntronMax 1, and --alignEndsType EndToEnd. After we obtained the mapping files, duplicate reads were removed using Picard (http://broadinstitute.github.io/picard/) with parameters MarkDuplicates and REMOVE_DUPLICATES = true. We calculated gene read counts using FeatureCounts (93) and converted the read counts to reads per kilobase of transcript per million mapped reads (RPKM) using the edgeR (94) package in the R language. DESeq2 (95) was used to obtain the differentially expressed genes between ADAR1 knockdown and negative control data sets. Only genes with fold change greater than 2 and adjusted *P* value below 0.05 were retained for further analysis. The WIG files of each RNA-Seq data set from sense strand and antisense strand were obtained through bamCoverage from deepTools (96) with the parameters “ignore duplicates” and “normalize using CPM.”

### Patient database bioinformatics.

For TCGA editing sites survival analysis, we downloaded the editing level of each editing site in GBM from Synapse (17); then Cox proportional hazard models and log-rank tests were used to determine the functional editing sites that were associated with patient survival (*P <* 0.05). For the gene survival analysis, we obtained the gene expression and patient information of GBM from the REMBRANDT data set, CGGA data set, and TCGA data set through the GlioVis web portal (http://gliovis.bioinfo.cnio.es) (97), and then performed survival analysis using the Cox proportional hazards model and log-rank tests for selected genes. The gene expression correlation analysis and differential expression analysis were also based on the previous data sets.

### Statistics.

All the statistical analyses are described in the figure legends. For survival analyses, the significance was calculated by log-rank (Mantel-Cox) test. For cell proliferation, 2-way ANOVA was used for analysis with Dunnett’s multiple-comparison test. For in vitro sphere formation analyses, pairwise tests were used for differences in stem cell frequencies. For the other analyses, Student’s *t* test was performed to assess the statistical significance between 2 groups. For comparison of more than 2 groups, 1-way ANOVA was used. Statistical analysis was performed with GraphPad Prism software.

### Study approval.

All procedures related to GBM tissues obtained from surgical resection performed with written informed consent at Duke University were in accordance with protocol 090401, which was approved by Institutional Review Board (Duke University, Durham, NC, USA). All murine experiments were performed under a protocol (s17096) approved by the UCSD Institutional Animal Care and Use Committee.

## Author contributions

LJ initiated and designed the study, acquired and analyzed the data, and wrote and revised the manuscript. YH designed the study, analyzed the RNA-Seq data, and revised the manuscript. CS performed experiments. QW provided experimental assistance for intracranial injections. RCG and BCP analyzed the data and revised the manuscript. GS, LJYK, GZ, ZQ, and ZZ revised the manuscript. XDF and JNR designed the study, analyzed the data, wrote and revised the manuscript, and provided study supervision and material support.

## Supplementary Material

Supplemental data

## Figures and Tables

**Table 2:**
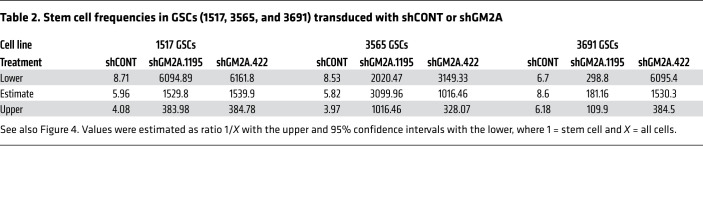
Stem cell frequencies in GSCs (1517, 3565, and 3691) transduced with shCONT or shGM2A

**Table 1:**
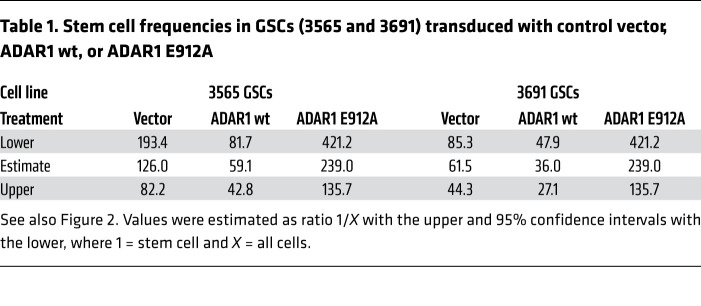
Stem cell frequencies in GSCs (3565 and 3691) transduced with control vector, ADAR1 wt, or ADAR1 E912A

## Version 1 02/08/2022

In-Press Preview

## Version 2 03/15/2022

Electronic publication
