# Supplementary material for: ADAR1-mediated RNA editing links ganglioside catabolism to glioblastoma stem cell maintenance
Source: J Clin Invest. 2022 Mar 15;132(6):e143397. doi: 10.1172/JCI143397 (PMC8920333; doi:10.1172/JCI143397)

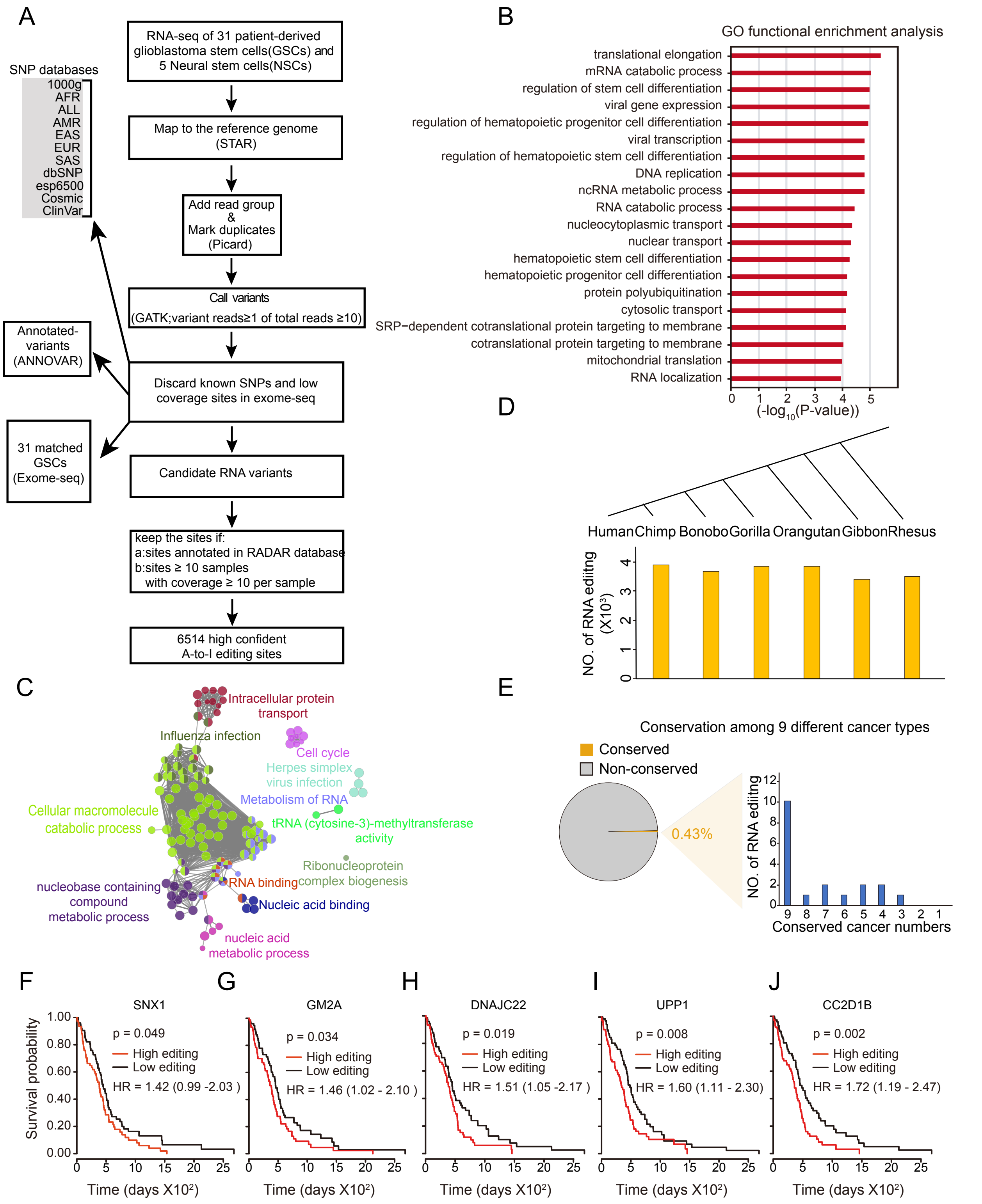

Supplemental Figure 1. RNA editome landscape of glioblastoma stem cells. Refers to Figure 1. (**A**) Workflow of RNA editing analysis. (**B**) Functional enrichment analysis of RNA editing sites. (**C**) Pathway enrichment bubble plot of genes marked by higher editing levels in GSCs. (**D**) Conservation analysis of RNA editing sites in different species. Bars represent the sum of conserved number of edits in the 7 designated species. (**E**) Conservation analysis of RNA editing sites in 9 cancer types (Bladder Urothelial Carcinoma, BLCA; Breast invasive carcinoma, BRCA; Cervical squamous cell carcinoma and endocervical adenocarcinoma, CESC; Colorectal carcinoma, CRC; Head and Neck squamous cell carcinoma, HNSC; Kidney Chromophobe, KICH; Kidney renal papillary cell carcinoma, KIRP; Low-Grade Glioma, LGG; and Uterine Corpus Endometrial Carcinoma, UCEC). (**F-J**) Kaplan-Meier survival curves of glioblastoma patients for editing level for indicated gene performed in 1L from TCGA data segregated by the median level. P-value was determined by log-rank test.

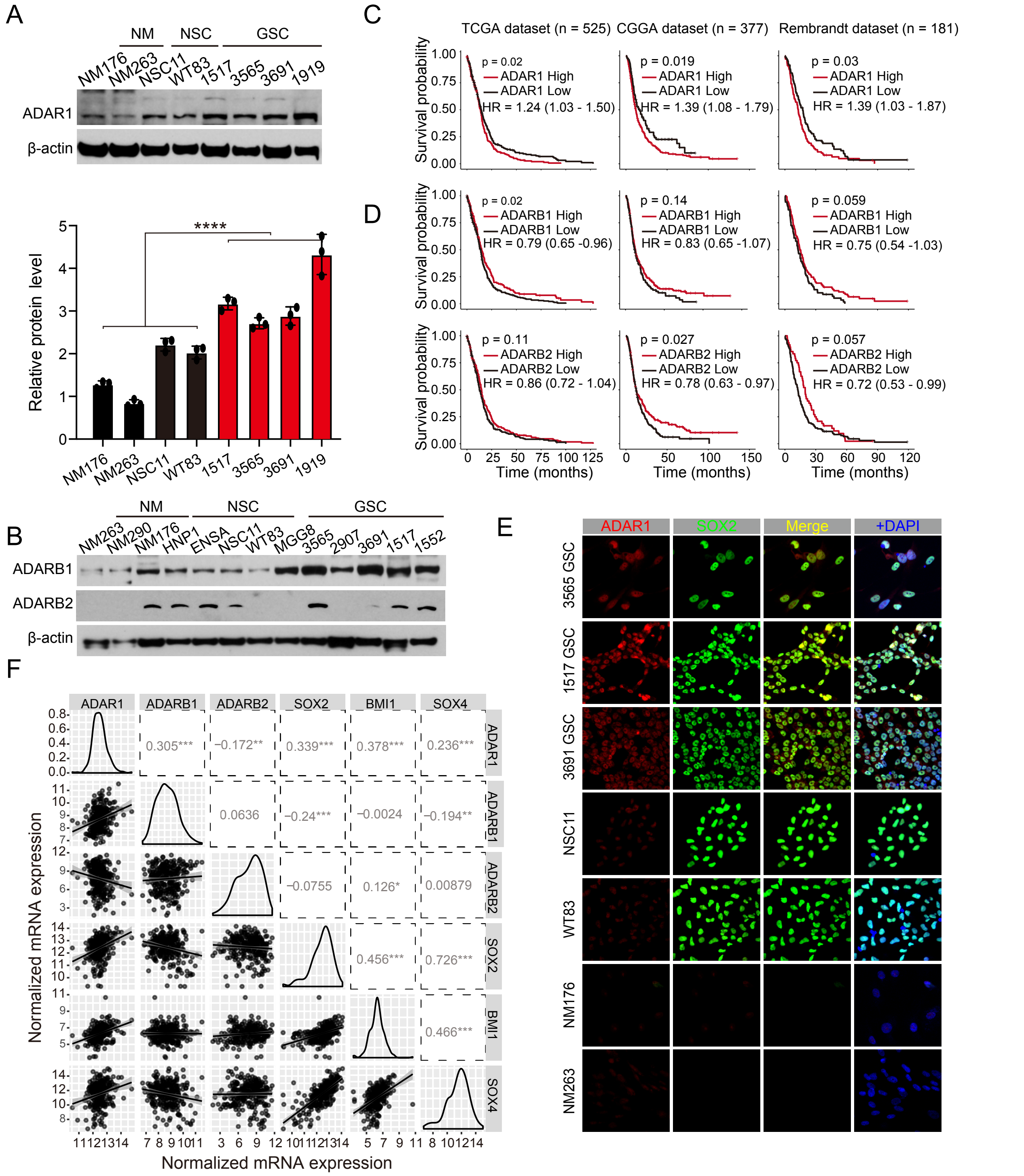

Supplemental Figure 2. ADAR1 levels are enriched in glioblastoma stem cells. Refers to Figure 2. (**A**) Western blots of ADAR1 in NMs (NM176 and NM263), NSCs (NSC11 and WT83), and GSCs (1517, 3565, 3691, and 1919). β-actin served as loading control (upper panel). ADAR1 protein levels were quantified by ImageJ (lower panel). Statistical significance was determined by ANOVA. N = 3. ****P < 0.0001. (**B**) Western blotting of ADARB1 or ADARB2 in NMs (NM176, NM293, and NM263), NSCs (HNP1, ENSA, NSC11, and WT83), and GSCs (MGG8, 3565, 2907, 3691, 1517, and 1552). β-actin served as loading control. (**C**) Kaplan-Meier survival curves of GBM patients based on ADAR1 mRNA in TCGA, CGGA, and Rembrandt databases segregated based on median expression. P-value was determined by log-rank test. (**D**) Kaplan-Meier survival curves of GBM patients based on ADARB1 (upper panel) or ADARB2 (lower panel) mRNA in TCGA, CGGA, and Rembrandt databases segregated based on median expression. P-value was determined by log-rank test. (**E**) Immunofluorescence analysis of ADAR1 and SOX2 in NMs (NM176 and NM263), NSCs (NSC11 and WT83) and GSCs (1517, 3565, and 3691). DAPI serves as a nuclear control. Scale bar: 50μm. (**F**) Pairwise correlation analysis between ADARs and 3 stem cell representative genes derived from the GBM TCGA. Plots indicate gene expression data. Correlation coefficient (R) values are shown.

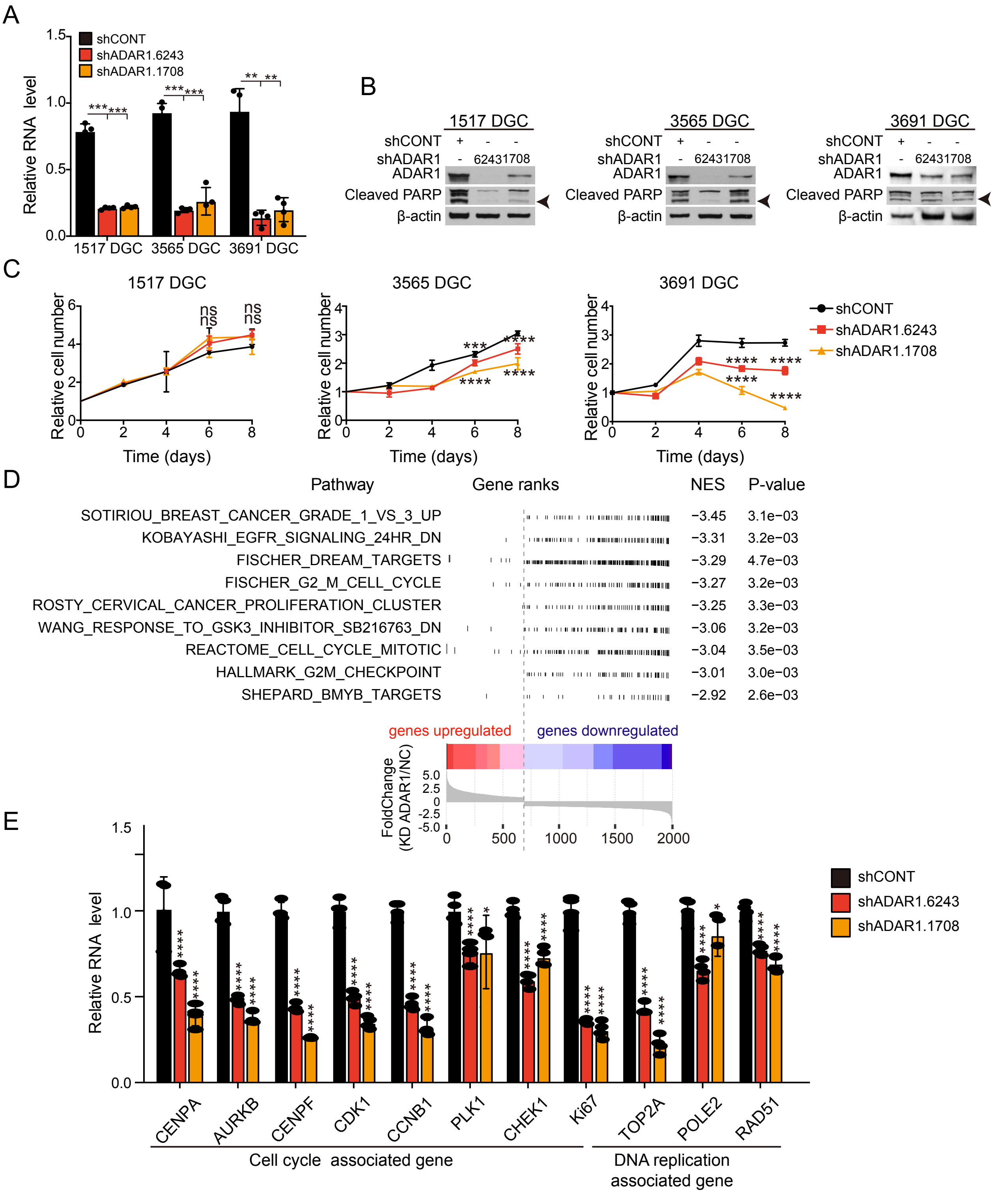

Supplemental Figure 3. ADAR1 is largely dispensable in differentiated glioblastoma cells, but regulates proliferation in glioblastoma stem cells. Refers to Figure 2. **(A)** ADAR1 mRNA expression of DGCs (1517, 3691, and 3565) transduced with shCONT or one of two, non-overlapping shADAR1s. N = 4. Quantitative data from four independent experiments are shown as the mean ± SD (error bars). Statistical significance was determined by ANOVA. **P < 0.01, ***P < 0.001. (**B**) Western blotting of ADAR1 and PARP of DGCs (1517, 3565, and 3691) transduced with shCONT or one of two, non-overlapping shADAR1s. β-actin served as loading control. Arrowheads indicate the Cleaved PARP. (**C**) Proliferation of DGCs (1517, 3565, and 3691) transduced with shCONT or one of two, non-overlapping shADAR1s determined by CellTiter-Glo. Quantitative data from five technical experiments are shown as the mean ± SD (error bars). Statistical significance was determined by two-way ANOVA with Dunnett's multiple comparisons test. ***P < 0.001, ****P < 0.0001. ns, not significant. (**D**) Downregulated gene signatures in GSCs transduced with shADAR1. P-values were empirically determined by sampling random gene sets. (**E**) The mRNA levels of cell cycle-associated genes and DNA replication-associated genes in 3691 GSCs transduced with shCONT or one of two, non-overlapping shADAR1s determined by RT-PCR. N = 4. Statistical significance was determined by ANOVA. *P < 0.05, ****P < 0.0001.

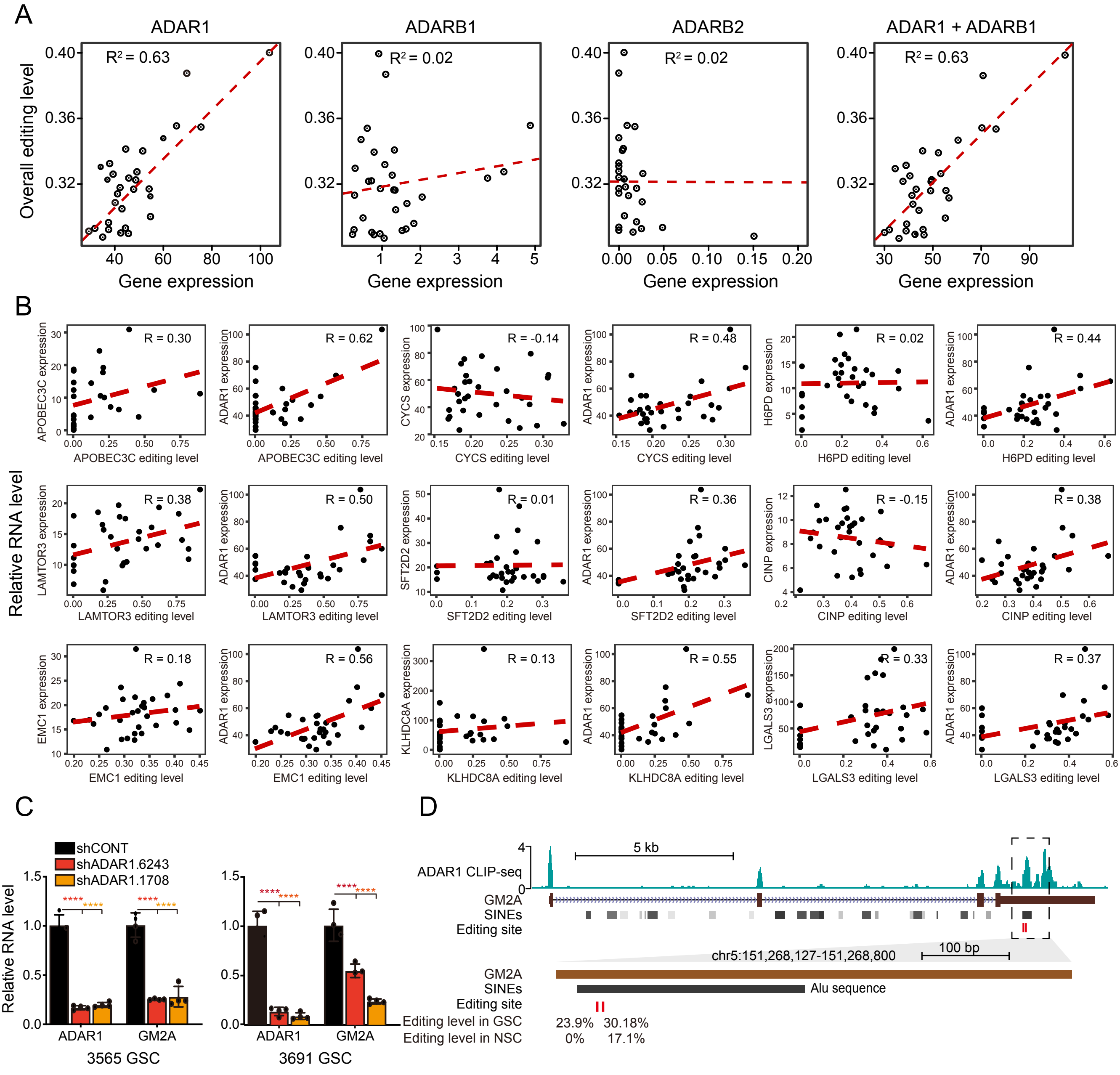

Supplemental Figure 4. ADAR1 is the dominant A-to-I editor in glioblastoma and regulates GM2A. Refers to Figure 3. (**A**) Correlation between expression levels of ADARs and overall editing level of high confident editing sites in GSCs. (**B**) Correlation between expression levels of ADAR1 and overall editing level of indicated genes and correlation between expression levels and overall editing levels of indicated genes in GSCs. (**C**) mRNA expression of ADAR1 and GM2A in GSCs (3565 and 3691) transduced with shCONT or shADAR1. Quantitative data from four independent experiments are shown as the mean ± SD (error bars). N = 4. Statistical significance was determined by ANOVA. ****P < 0.0001. (**D**) The editing pattern of GM2A. The ADAR1 binding peaks across GM2A mRNA (upper panels). The lower panels indicated the position of editing sites of GM2A within SINEs and the editing level in GSC and NSC.

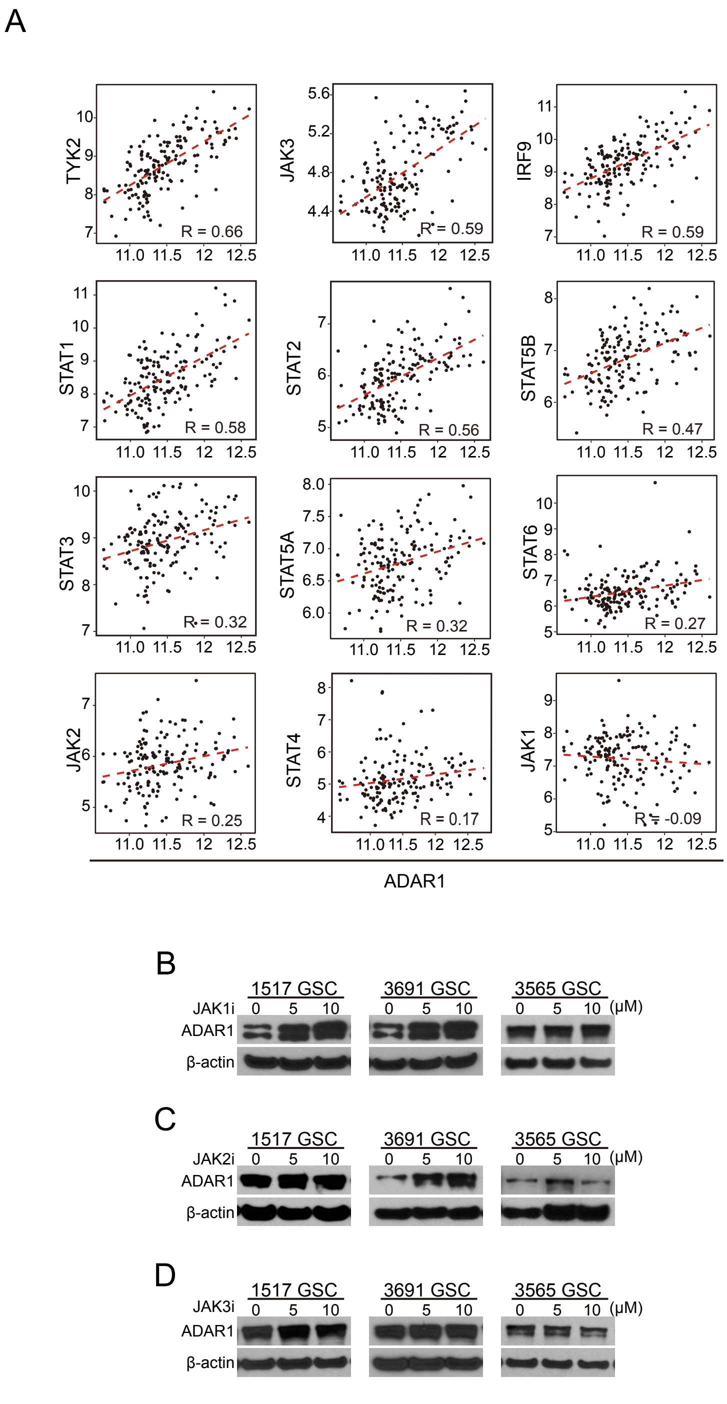

Supplemental Figure 5. Relationship between ADAR1 and JAK/STAT family members. Refers to Figure 7. (A) Correlation between ADAR1 and 12 JAK-STAT Pathway representative genes Plots indicate gene expression data from TCGA datasets. Correlation coefficient values are shown. (B) Western blotting of ADAR1 in GSCs (1517, 3691, and 3565) with different concentrations of a JAK1 inhibitor. β-actin served as loading control. (C) Western blotting of ADAR1 in GSCs (1517, 3691, and 3565) with different concentrations of a JAK2 inhibitor. β-actin served as loading control. (D) Western blotting of ADAR1 in GSCs (1517, 3691, and 3565) with different concentrations of a JAK3 inhibitor. β-actin served as loading control.

Supplementary Table 1: Antibodies used in this study

All the antibodies used in this study are listed

Antigen	Host	Vendor	Catalogue#	Dilution (WB)	Dilution (IF)
ADAR1	rabbit	cell signaling technology	14175s	1:1000	
ADAR1	mouse	abcam	ab88574		1:100
GFAP	mouse	sigma	G3893	1:1000	
SOX2	goat	R&D Systems	AF2018		
β-actin	mouse	sigma	A5316	1:20000	
GM2A	rabbit	Proteintech	10864-2-AP		
GM2	rabbit	Matreya	1951		1:50
Lamp2	mouse	novus	NBP2-22217		1:100
ADARB1	rabbit	Proteintech	22248-1-AP		
ADARB2	mouse	santa	sc-73410	1:500	
PARP	rabbit	cell signaling technology	9532S	1:1000	
Caspase3	rabbit	cell signaling technology	9664S	1:1000	

Supplementary Table 2: DNA oligos used in this study
All the DNA oligos used in this study are listed

**Primer oligos for quantifying gene expression**		
**Target**	**Strand**	**Sequence(5'->3')**
ADAR1	Forward	TCAGCGGGCTGTTAGAATATG
	Reverse	AAACTCTCGGCCATTGATGA
GM2A	Forward	TCGTTCCTGGAAATGTGACC
	Reverse	AGCCACCTCCTTCTCCAAAA
ACTB	Forward	TCCCTGGAGAAGAGCTACGA
	Reverse	AGCACTGTGTTGGCGTACAG
CENPA	Forward	TTCCTCCCATCAACACAGTCG
	Reverse	CACACCACGAGTGAATTTAACAC
AURKB	Forward	AAGGAGAACTCCTACCCCTGG
	Reverse	ACAAGTGCAGATGGGGTGAC
CENPF	Forward	GTTCCAGGCGAGTCAGATCA
	Reverse	TTCTCTGGCCTTTTGCTCAG
CDK1	Forward	AGGAAGGGGTTCCTAGTACTGC
	Reverse	TGGAATCCTGCATAAGCACA
CCNB1	Forward	GTGGATGCAGAAGATGGAGC
	Reverse	CCGACCCAGTAGGTATTTTGG
PLK1	Forward	GGAAGCCCCTCACAGTCC
	Reverse	CGACCACCTCACCTGTCTCT
CHEK1	Forward	CATCAGCAAGAATTACCATTCCA
	Reverse	CACCACCTGAAGTGACTCGG
Ki67	Forward	TGAGCCTGTACGGCTAAAACA
	Reverse	TCAGTTGACTTCCTTCCATTCTG
TOP2A	Forward	ACCCAAGAGCTTTGGATCAAC
	Reverse	GGACTTGGGCCTTAAACTTCA
POLE2	Forward	CCTCACAGAAGCTCTTCAGTCTATC
	Reverse	TTCCACCACAGATCGTTCAA
RAD51	Forward	CTGAGGGTACCTTTAGGCCAG
	Reverse	TGGGTCTGGTGGTCTGTGTT
GM2A	Forward	TGTTTACAAACTCAGGTACCCG
	Reverse	ACTCTCCAACTGACTCCAGTC
HEXA	Forward	GGGGCTTGCTGTTGGATAC
	Reverse	TACCAGATGCCAGTGGAACA
HEXB	Forward	GTTGATGACCAGTCTTTCCCAT
	Reverse	CACCATACGGACATCATTTGG
18s rRNA	Forward	GAAGGGCACCACCAGGAGT
	Reverse	CAGACAAATCACTCCACCAA
**Primers for constructing expression plasmids**		
**Target**	**Strand**	**Sequence(5'->3')**
ADAR1-wt	Forward	attGCTAGC atgaatccgcggcaggggta
	Reverse	attGCGGCCGCtactgggcagagataaaagttcttttcct
ADAR1-E912A	Forward	ctgtcaatgactgccatgcagcaataatctcccggagaggcttc
	Reverse	gaagcctctccgggagattattgctgcatggcagtcattgacag
**DNA oligos for shRNA**		
**Target**	**TRC number**	**Sequence(5'->3')**
ADAR1	TRCN0000050788	CCGGGCCCACTGTTATCTTCACTTTCTCGAGAAAGTGAAGATA ACAGTGGGCTTTTTG
	TRCN0000050790	CCGGGCTGTTAGAATATGCCCAGTTCTCGAGAACTGGGCATAT TCTAACAGCTTTTTG
GM2A	TRCN0000008694	CCGGCCTTAACTTCTGTGACTAATTCTCGAGAATTAGTCACAGA AGTTAAGGTTTTT
	TRCN0000008697	CCGGGATCCCATGCACAGACTACATCTCGAGATGTAGTCTGTG CATGGGATCTTTTT
HEXA	TRCN0000029284	CCGGCCCAGTCTCAATAATACCTATCTCGAGATAGGTATTATTG AGACTGGGTTTTT
	TRCN0000029286	CCGGCGTCCTTTACCCGAACAACTTCTCGAGAAGTTGTTCGGG TAAAGGACGTTTTT
HEXB	TRCN0000218458	GTACCGGGTCCGTATGGTGATTGAATATCTCGAGATATTCAAT CACCATACGGACTTTTTTG
	TRCN0000231056	CCGGGGATATTATTGCAACCATAAACTCGAGTTTATGGTTGCAA TAATATCCTTTTTG

Full unedited gel for Suppl Figure 2A

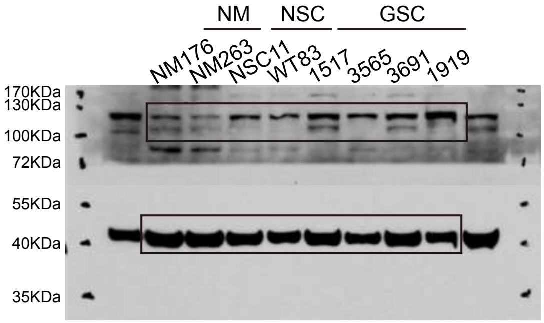

Full unedited gel for Suppl Figure 2B

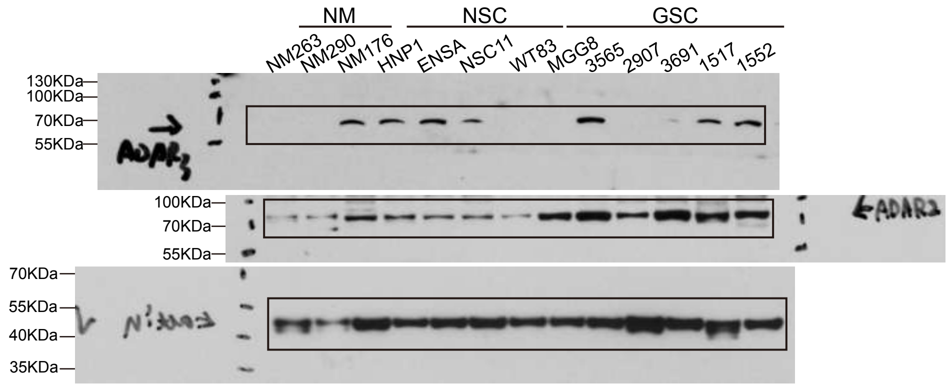

Full unedited gel for Suppl Figure 3B

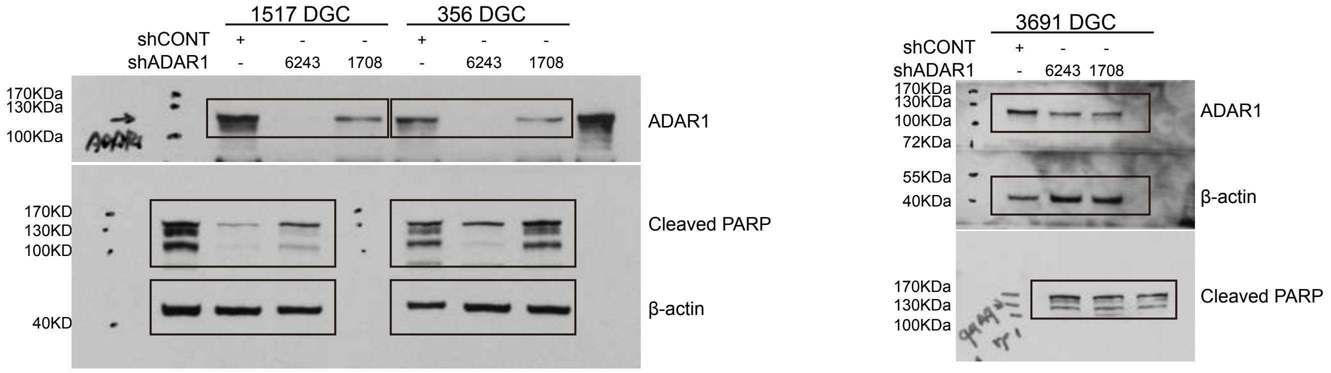

Full unedited gel for Suppl Figure 5B

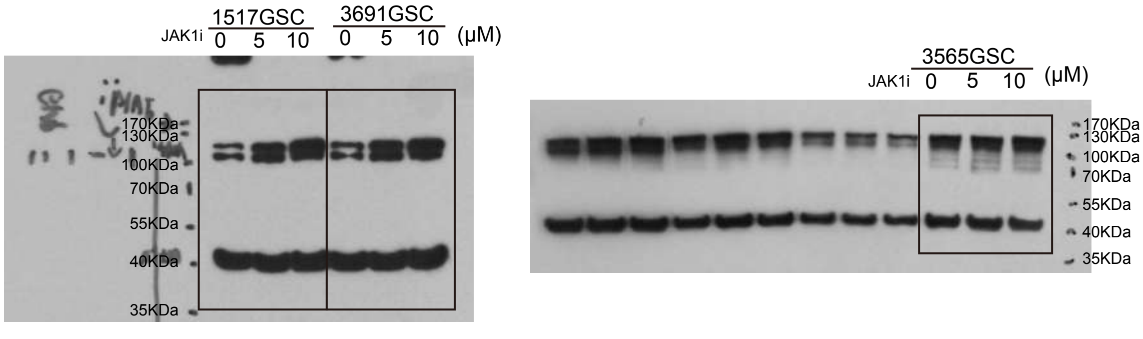

Full unedited gel for Suppl Figure 5C

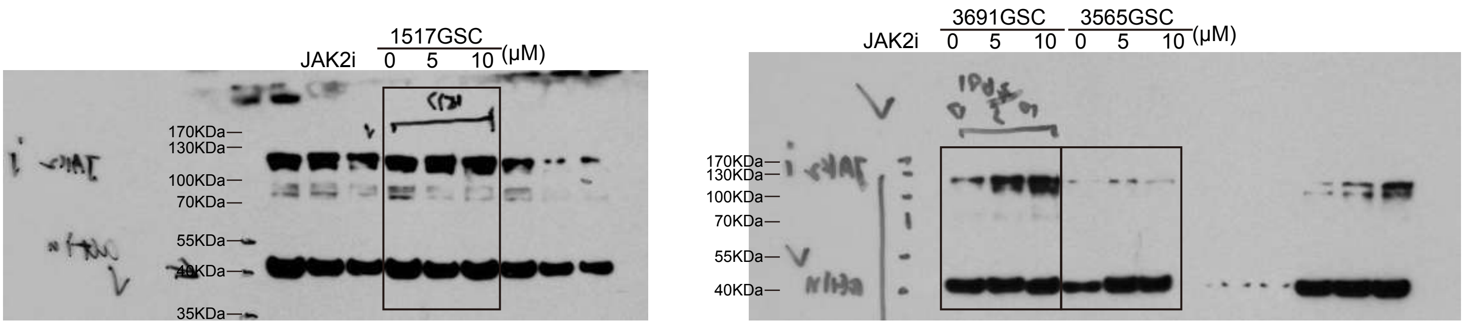

Full unedited gel for Suppl Figure 5D

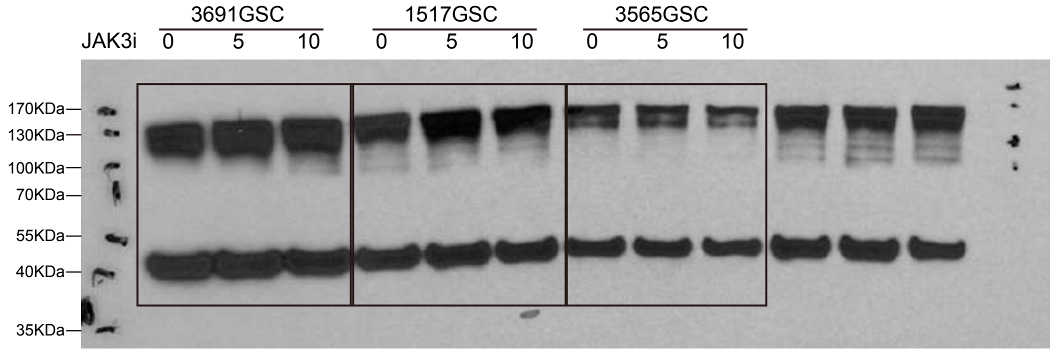

Full unedited gel for Figure 2B

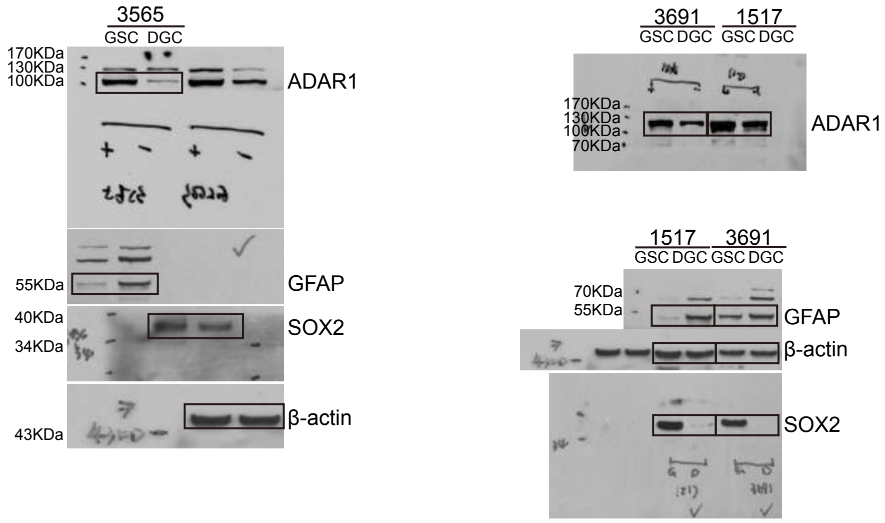

Full unedited gel for Figure 2F

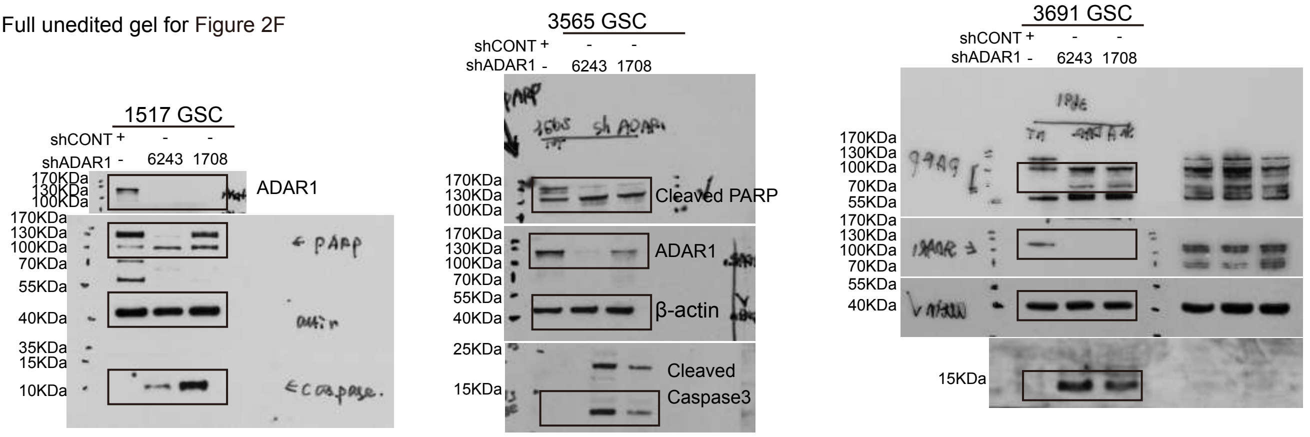

Full unedited gel for Figure 2K

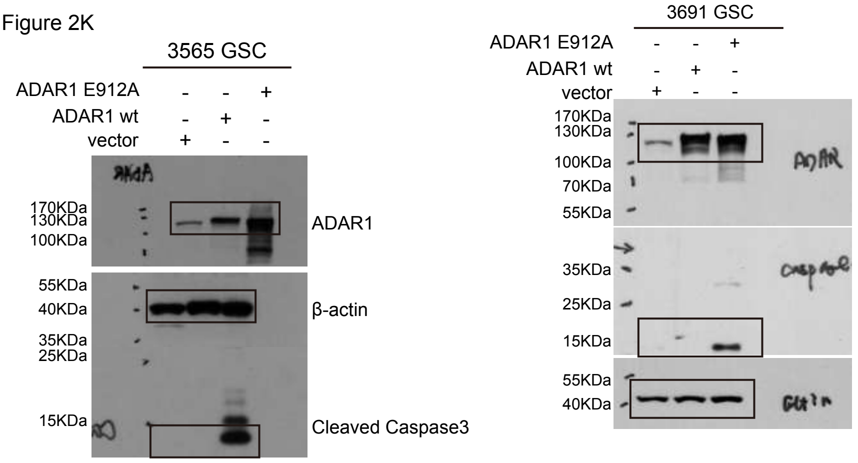

Full unedited gel for Figure 3D

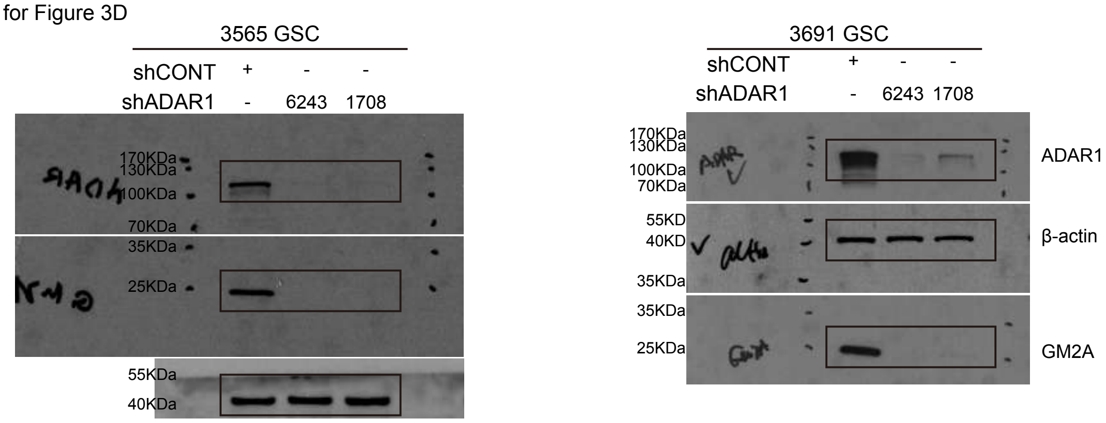

Full unedited gel for Figure 3F

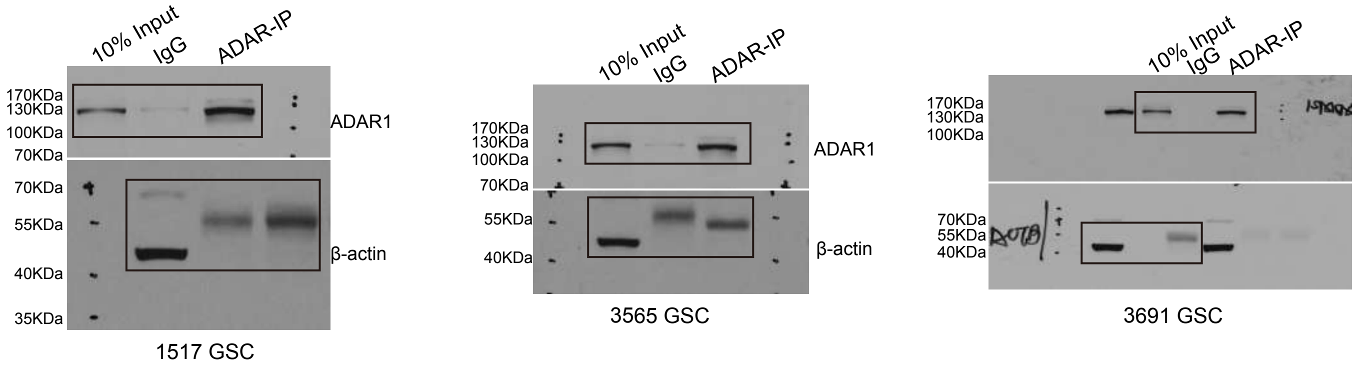

Full unedited gel for Figure 3G

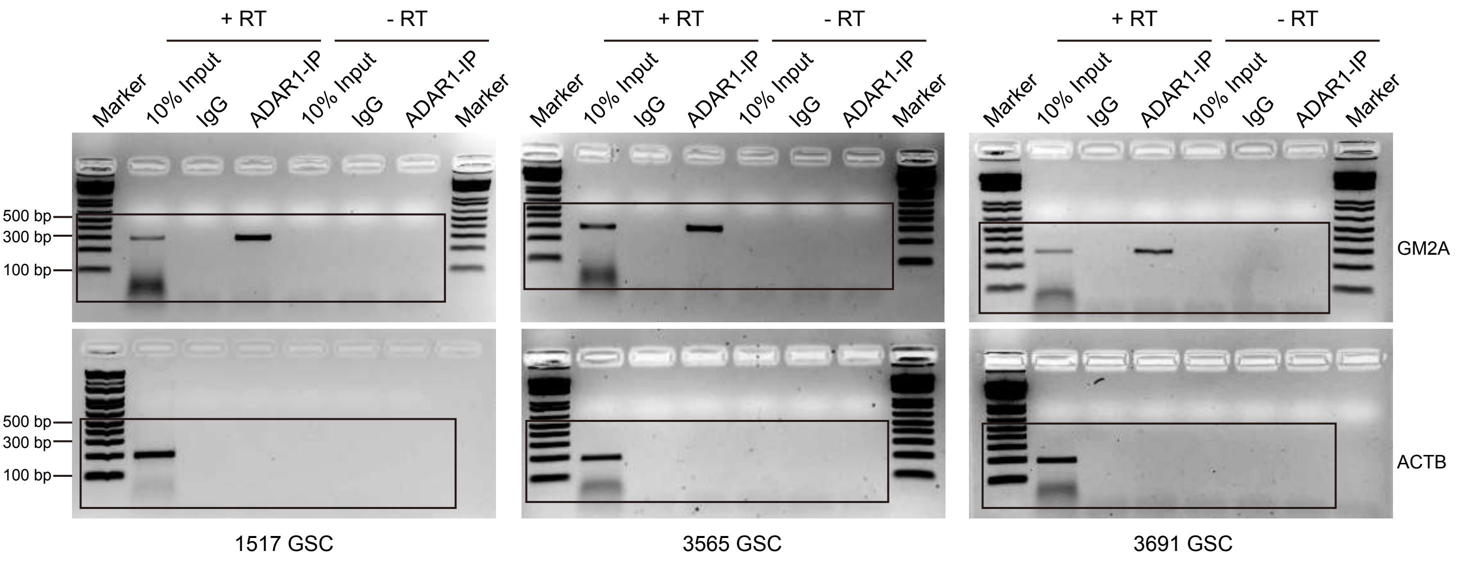

Full unedited gel for Figure 3I

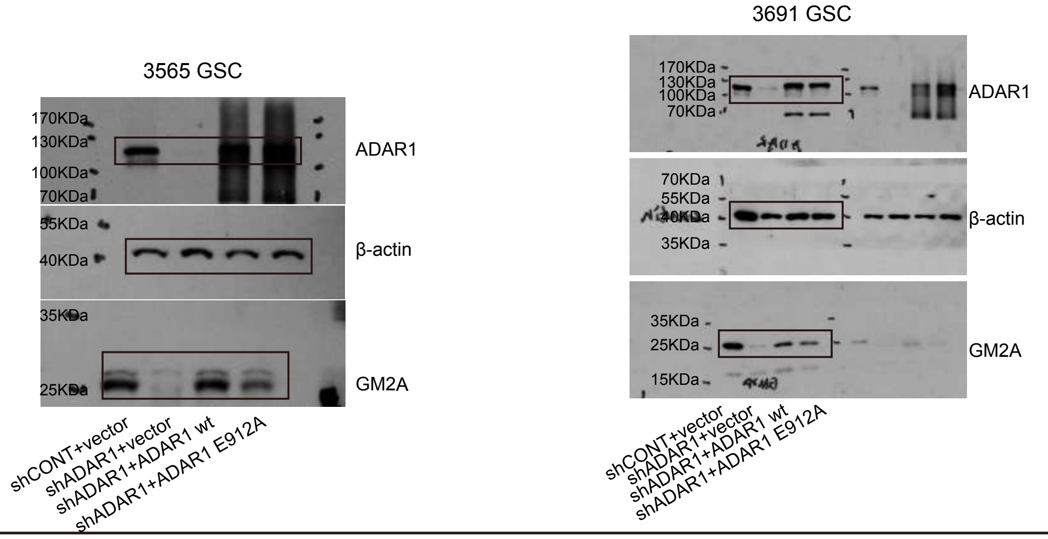

Full unedited gel for Figure 4C

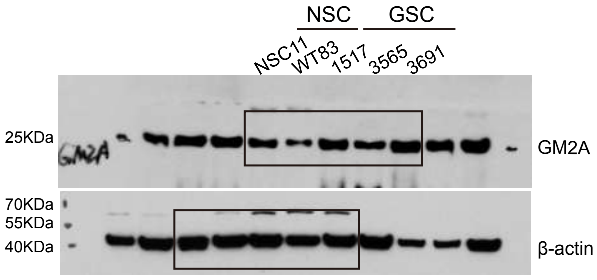

Full unedited gel for Figure 4D

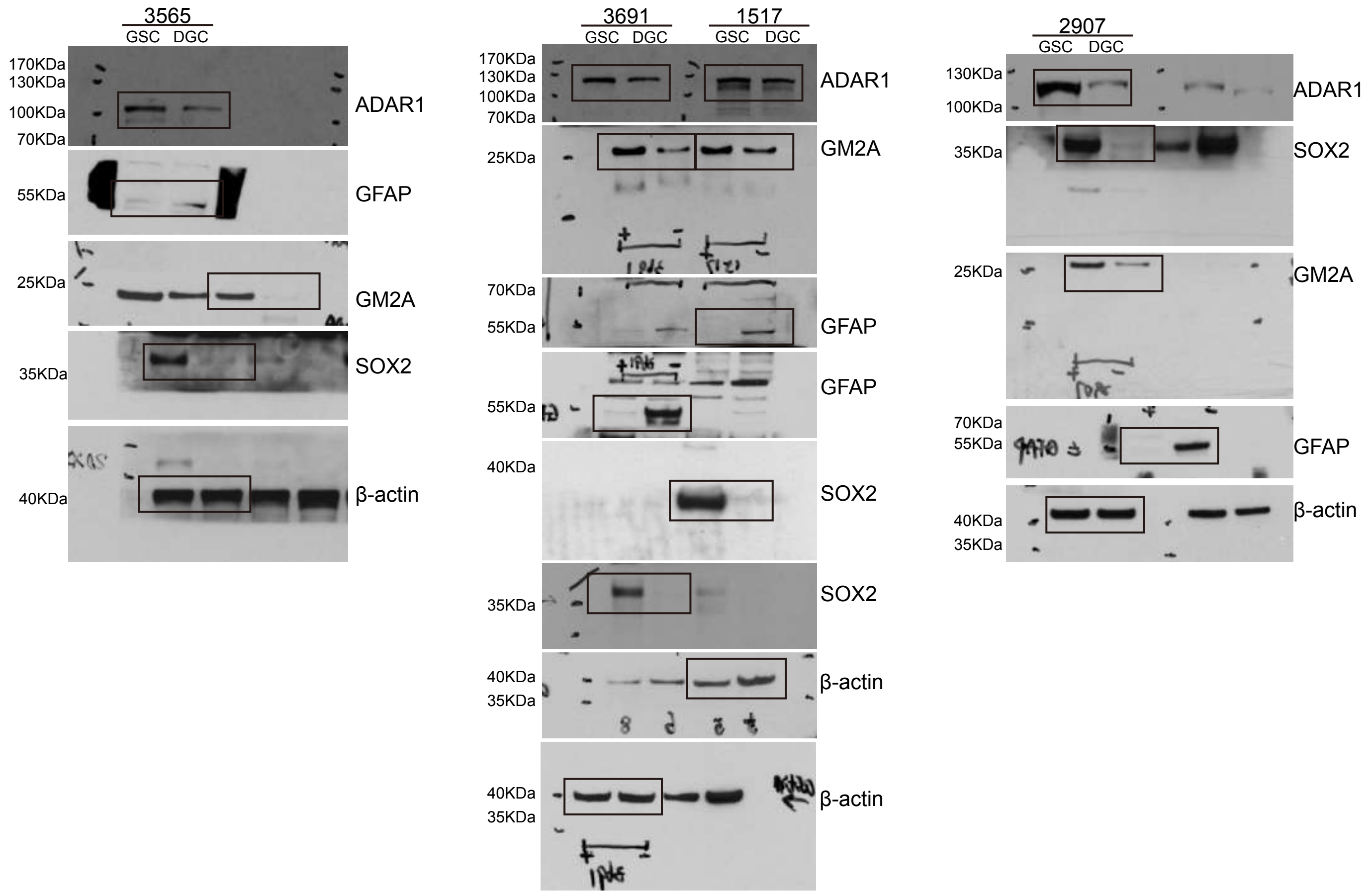

Full unedited gel for Figure 4F

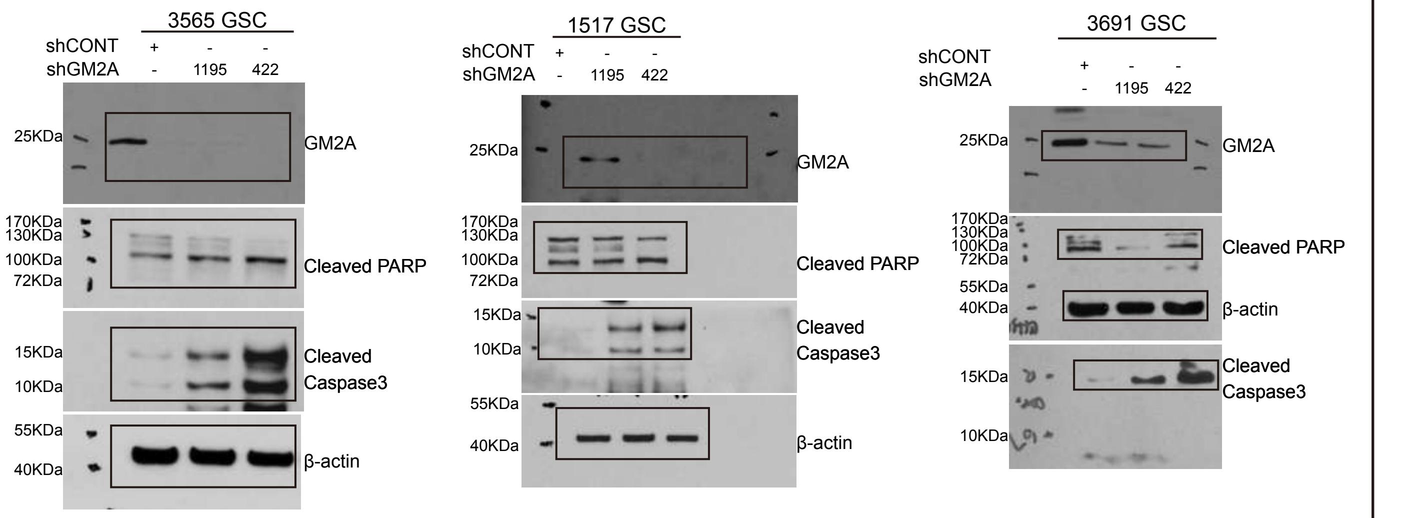

Full unedited gel for Figure 7D

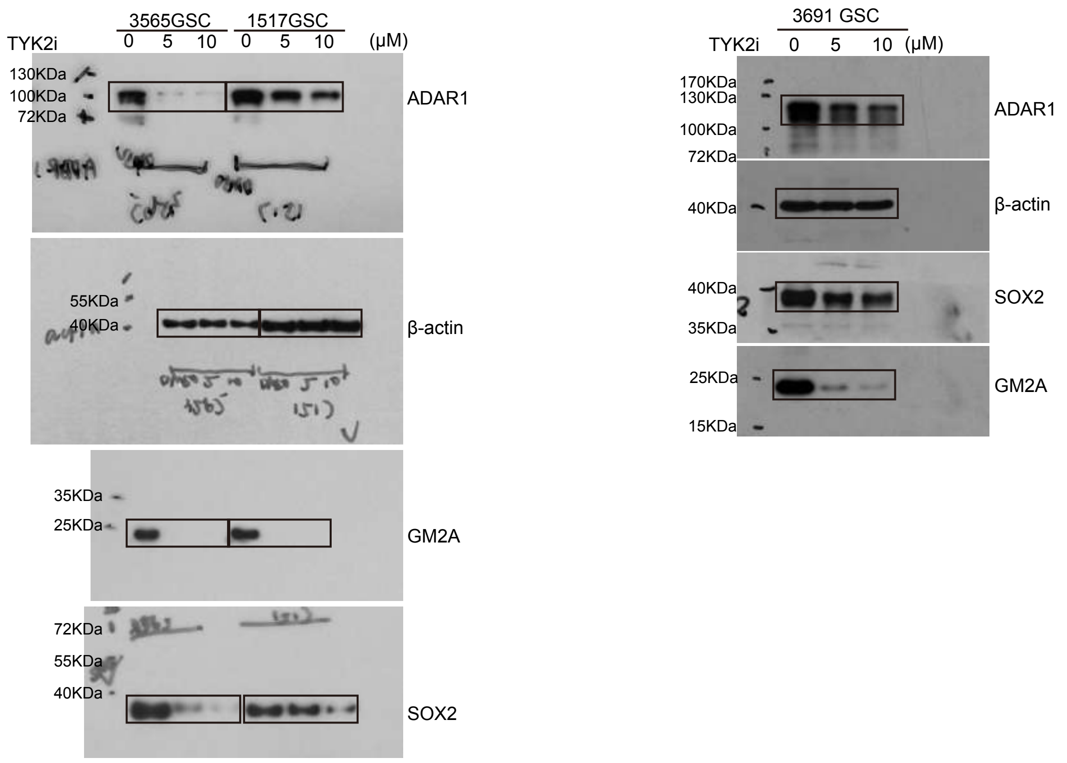

Supplement: Supplemental data [file jci-132-143397-s190.pdf]
